# Cancer‐Specific RNA Modifications in Tumour‐Derived Extracellular Vesicles Promote Tumour Growth

**DOI:** 10.1002/jev2.70083

**Published:** 2025-05-06

**Authors:** Yuya Monoe, Kentaro Jingushi, Kohei Taniguchi, Kensuke Hirosuna, Jun Arima, Yosuke Inomata, Yoshiaki Takano, Hiroki Hamamoto, Kazumasa Komura, Tomohito Tanaka, Hiroaki Hase, Sang‐Woong Lee, Kazutake Tsujikawa

**Affiliations:** ^1^ Laboratory of Molecular and Cellular Physiology, Graduate School of Pharmaceutical Sciences Osaka University Suita Osaka Japan; ^2^ Center for Medical Research & Development, Division of Translational Research Osaka Medical and Pharmaceutical University Takatsuki Osaka Japan; ^3^ Department of Regenerative Science Okayama University Graduate School of Medicine, Dentistry and Pharmaceutical Sciences Okayama Japan; ^4^ Department of General and Gastroenterological Surgery Osaka Medical and Pharmaceutical University Takatsuki Osaka Japan

**Keywords:** 5′‐half‐GlyGCC, colorectal cancer, extracellular vesicles, inflammatory cytokines, RNA modification

## Abstract

RNA modifications are crucial in cellular processes, and their dysregulation is linked to diseases like cancer. Extracellular vesicles (EVs) contain various RNAs and might be susceptible to modifications, but detecting these modifications has been challenging due to the small amount of RNA in EVs. We successfully detected 22 RNA modifications in EVs using a proprietary ultra‐HPLC MS/MS system. We identified reduced levels of N6‐methyladenosine (m6A) in EVs derived from colon cancer tissues, which correlated with cancer recurrence. Increasing m6A levels via m6A demethylase Alkbh5 knockout suppressed the tumour‐promoting effects of colorectal cancer EVs. Mechanistically, colorectal cancer‐derived EVs increased tumour necrotic factor α and interleukin‐6 secretion by macrophages via Toll‐like receptor 8 in an m6A‐dependent manner, promoting cancer cell proliferation. RNA‐sequencing analysis showed that the levels of 5′‐half‐tRNA fragment (5′‐half)‐GlyGCC as well as those of m6A‐modified 5′‐half‐GlyGCC were higher and lower, respectively, in colorectal cancer EVs than in normal colon tissue EVs. Cancer‐derived EVs containing 5′‐half‐GlyGCC significantly promoted tumour growth, which was impeded by macrophage depletion. These findings provide evidence that cancer‐specific RNA modifications are present in EVs, promoting tumour progression by regulating immune cells.

## Graphical abstract

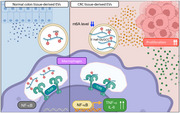

## 1 Introduction

To date, over 170 types of RNA modifications have been identified in various organisms (Liu et al. 2024). These modifications are critical regulators of cell biology, influencing RNA biogenesis, transport, function and metabolism (Cui et al. 2022). Alterations in RNA modification levels have been observed in various diseases (Frye et al. 2018) and are associated with cancer cell proliferation, metastasis and immune evasion (Barbieri and Kouzarides 2020, Yang et al. 2021, Monoe et al. 2023, Kogaki et al. 2021). However, most studies have focused on the expression of RNA modification writers, readers and erasers, with few directly exploring the effects of these modifications on cancer progression and metastasis (Orsolic et al. 2023). Colorectal cancer (CRC) is the third most common and second deadliest cancer worldwide (Abedizadeh et al. 2023). The tumour immune microenvironment is closely associated with CRC development (Jang et al. 2021). Systemic inflammation is a marker of poor prognosis in approximately 20%–40% of CRC cases (Schmitt and Greten 2021).

m6A (N6‐methyladenosine), m5C (5‐methylcytosine) and Ψ (pseudouridine) modifications are frequently observed on mammalian RNAs and suppress host responses to RNA via Toll‐like receptors (TLRs). The low frequency of RNA modifications in viruses and bacteria indicates that TLRs may distinguish between self and non‐self RNA modifications (Karikó et al. 2005). Recently, m6A has been implicated in tumour immunoregulation, with divergent m6A methylation patterns observed within the tumour microenvironment (Liu et al. 2022). However, the regulation of m6A in tumour‐infiltrating immune cells remains unknown.

Extracellular vesicles (EVs) have emerged as mediators of cell–cell interactions and are released by almost all cell types. EVs are broadly classified into small (<200 nm) and large (≥200 nm) categories based on their size (Jeppesen et al. 2023). Cancer cell‐derived EVs promote tumorigenesis and metastasis by acting on cancer cells, immune cells, fibroblasts and endothelial cells (Urabe et al. 2017, Kosaka et al. 2016, Möller and Lobb 2020, Becker et al. 2016, Paskeh et al. 2022). Given that EVs contain various intracellular RNAs, it is expected that they also harbour modified RNAs. However, due to the small amount of RNA in EVs, the presence and pathological significance of RNA modifications have not been determined. We previously analysed 46 RNA modifications using ultra‐high‐performance liquid chromatography‐unispray‐tandem mass spectrometry (UHPLC‐UniSpray‐MS/MS) and successfully captured the landscape of RNA modifications characteristic of lung and ovarian cancer tumour tissue (Monoe et al. 2023, Kogaki et al. 2021). The high sensitivity of this system allows for the analysis of trace RNA modifications in EVs.

We hypothesised that cancer cell‐derived EVs harbour modified RNA that modulates tumour immunoregulation. Here, we identified CRC‐specific RNA modifications in EVs that promote tumour growth by enhancing tumour necrosis factor (TNF)‐α and interleukin (IL)‐6 secretion by macrophages. Decreased m6A levels on 5′‐half‐tRNA fragment (5′‐half)‐GlyGCC in EVs were identified as determinants of this effect and subsequent tumour progression, acting via Toll‐like receptor 8 (TLR8). Furthermore, we showed that cancer‐derived EVs harbour modified RNAs and modulate tumour immunoregulation by macrophages.

## 2 Materials and Methods

### 2.1 Clinical Specimens

Seventy‐six cancer tissues and adjacent normal colon tissues were obtained from patients who underwent resection at Osaka Medical and Pharmaceutical University Hospital (Japan). Written informed consent was obtained from each patient, and the study was approved by the Ethics Review Board of the Osaka Medical and Pharmaceutical University Hospital (2305 and 2808) and Osaka University Graduate School of Pharmaceutical Sciences (Yakuhito30‐17‐3) and conducted in accordance with the Declaration of Helsinki. The clinical and histopathological data of the specimens are presented in Table S1.

### 2.2 Cell Culture

Human peripheral blood mononuclear cells (PBMCs) and human peripheral blood CD14+ monocytes obtained from Precision for Medicine (Bethesda, MD, USA) were cultured in RPMI‐1640 medium (Wako, Osaka, Japan) supplemented with 10% foetal bovine serum (FBS; Thermo Fisher Scientific, Waltham, MA, USA), 1 mM sodium pyruvate (Thermo Fisher Scientific), 1× nonessential amino acids (Thermo Fisher Scientific), 2 mM L‐glutamine (Thermo Fisher Scientific), 0.1 mg/mL kanamycin (Wako), 100 U/mL penicillin G (Wako) and 0.1 mg/mL streptomycin (Wako). The human colorectal cancer cell line HT29, normal colon cell line CCD‐841‐CoN and human monocyte cell line THP‐1 were obtained from the American Type Culture Collection (ATCC, Manassas, VA, USA). The murine colorectal cancer cell line colon26 was obtained from the Japanese Collection of Research Bioresources Cell Bank (JCRB, Osaka, Japan). These cells were cultured in RPMI‐1640 medium (Wako) supplemented with 10% FBS (Thermo Fisher Scientific) and 0.1 mg/mL kanamycin. The human colorectal cancer cell line SW620 (ATCC) and murine macrophage cell line RAW264.7 (ATCC) were cultured in DMEM (Wako) supplemented with 10% FBS (Thermo Fisher Scientific) and 0.1 mg/mL kanamycin (Wako).

### 2.3 Preparation of Macrophages

CD14+ monocytes were cultured in a medium supplemented with 20 ng/mL human GM‐CSF (Wako) for 48 h to differentiate into macrophages. THP‐1 cells were cultured in medium supplemented with 50 nM phorbol 12‐myristate 13‐acetate (PMA, Wako, CAS No. 16561‐29‐8) for 24 h to differentiate into macrophage‐like cells (dTHP‐1 cells). The cells were incubated in a fresh medium without PMA for 24 h before use.

### 2.4 Establishment of Alkbh5 Knockout Cells

Two gRNAs targeting 5′‐CACCGCTCAAGTCCATGACGTCCC‐3′ and 5′‐CACCGAAGAAGTACTTGTTGCGCAG‐3′ were inserted into the px330‐U6‐Chimeric_BB_CBh‐hSpCas9 vector (Addgene, Watertown, MA, USA) to establish Alkbh5 knockout cells. HT29 and Colon26 cells (8 × 10^4^ cells) were seeded in a 12‐well plate, and the next day, the medium was replaced with Opti‐MEM (Wako). Next, 500 ng of gRNA‐inserted Cas9 vector and 500 ng of puromycin‐resistant gene‐encoding pGL4.84 vector (Addgene) were mixed with 3 µL of Lipofectamine 2000 (Invitrogen) and co‐transfected into cells. Twenty‐four hours after transfection, the medium was replaced with RPMI medium supplemented with puromycin (1 µg/mL) to select transfected cells. After puromycin selection, surviving cells were cloned by limiting dilution. Clones were screened by immunoblotting with an anti‐Alkbh5 antibody (1:2000, Proteintech), and m6A levels were compared with those in control cells.

### 2.5 Animal Experiments

To establish allograft models, male BALB/c mice (aged 6–7 weeks) were purchased from SHIMIZU Laboratory Co., Ltd. Animals were kept under 12‐h light–dark cycles at 22°C–24°C. The mice were subcutaneously inoculated with 1 × 10^6^ colon26 cells. Six days after inoculation, PBS or EVs (4.5 × 10^8^ particles in 100 µL PBS) were intraperitoneally administered every day from Days 1–6. For the macrophage‐depleted mouse experiment, clodronate liposomes (HYGIEIA BIOSCIENCE) were administered intraperitoneally 2 days before EVs administration. Subsequently, PBS or EVs (4.5 × 10^8^ particles) were administered intraperitoneally from Days 1 to 6. Tumour volume was calculated as follows: (tumour length × tumour width^2^)/2. At the endpoint, tumours were resected, and frozen tumour tissues were subjected to immunohistochemistry.

All animal experiments were approved by the Animal Experimentation Committee of the Graduate School of Pharmaceutical Science at Osaka University (Douyaku R03‐14).

### 2.6 EV Isolation

Tissue samples (∼100 mg) were immediately immersed in 5 mL DMEM (Wako) without FBS at 37°C for 2 h. The tissue culture supernatant was centrifuged at 2000 × *g* for 30 min to remove cell debris and then centrifuged again at 10,000 × *g* for 30 min. The precipitate was recovered using 1 mL of PBS and collected as large Te‐EVs. The supernatant was subjected to ultracentrifugation (Optima L‐90K; SW55 rotor; Beckman Coulter, Pasadena, CA, USA) at 100,000 × *g* for 90 min. The pellets were washed with PBS at 100,000 × *g* for 90 min and recovered as small Te‐EVs in 1 mL of PBS.

For EV isolation from cell culture‐conditioned medium (CM), HT29, SW620, CCD‐841‐CoN and colon26 cells were seeded in five 10‐cm dishes with 8 mL of RPMI‐1640 medium supplemented with EV‐free FBS (Thermo Fisher Scientific) and incubated for 96 h. The CM was centrifuged at 2000 × *g* for 30 min to remove cell debris, and the collected supernatant was centrifuged at 10,000 × *g* for 30 min. The precipitate was collected as large EVs, and the supernatant was subjected to ultracentrifugation (Optima L‐90K; SW28 rotor; Beckman Coulter) at 100,000 × *g* for 90 min. The pellets were washed with PBS at 100,000 × *g* for 90 min and recovered as small Te‐EVs with 1 mL of PBS.

Purification of collected EVs from HT29 and SW620 CM was performed using qEV35 original column according to the manufacturer's instructions (Izon Science Ltd., Christchurch, UK).

EV size and concentration were determined using qNano Gold (Izon) with NP150 and NP400 nanopores, according to the manufacturer's instructions (Izon Science Ltd., Christchurch, UK). All EV samples and calibration particles (Izon Science Ltd.) were measured at 47.0 mm stretch and 0.5 kPa, with a voltage of 0.5–0.8 V. Data were analysed using the Izon Control Suite Software (V3.3.2.2001).

### 2.7 Transmission Electron Microscopy (TEM)

TEM was performed according to the method described by Cecilia et al. (Lässer et al. 2012). Briefly, EV samples (1 µg) were placed on a Formvar carbon‐coated nickel grid for 1 h, fixed with 2% paraformaldehyde, and observed under a transmission electron microscope (HT7800; Hitachi, Tokyo, Japan).

### 2.8 Western Blotting

Cells and EVs were lysed using Laemmli sodium dodecyl sulfate (SDS) sample buffer with or without 2‐mercaptoethanol. Protein samples (10 µg of cell lysate and 3 µg of EVs lysate) were heat‐denatured, separated on a 10%–12% gel using SDS‐polyacrylamide gel electrophoresis (PAGE), and then transferred onto a polyvinylidene difluoride (PVDF) membrane using a BioRad semi‐dry transfer system (1 h, 12 V, BioRad, Hercules, CA, USA). The membranes were blocked with 3% BSA/TBS with 0.1% Tween 20 (TBS‐T) for 1 h and then probed with the following primary antibodies: anti‐Alkbh5 (1:2000, Protein Tech), anti‐β‐actin (1:1000, Wako), anti‐phospho‐p65(Ser536) (1:500, Cell Signalling Technology), anti‐p65 (1:1000, CST), anti‐TLR3 (1:500, Santa Cruz Biotechnology, Inc.), anti‐TLR8 (1:500, Santa Cruz), anti‐human CD9 (1:1000, COSMO BIO), anti‐human CD63 (1:1000, COSMO BIO) anti‐mouse CD9 (1:1000, BioLegend) and anti‐mouse CD63 (1:1000, BioLegend). The membranes were then incubated with horseradish peroxidase (HRP)‐conjugated secondary antibodies against mouse IgG (1:5000, CST), rabbit IgG (1:5000, CST) and rat IgG (1:5000, CST), followed by detection using an enhanced chemiluminescence (ECL) Western blotting detection reagent (GE Healthcare). Chemiluminescence was detected using an Amersham Imager 680 (GE Healthcare).

### 2.9 RNA Isolation From EVs, Cells and Tissues

RNA contained in EVs, cells, and tissues was isolated using the miRNeasy mini kit (QIAGEN), following the manufacturer's protocol. RNA was extracted from small EVs (5 × 10^8^ particles), large EVs (5 × 10^7^ particles), cells (1 × 10^6^ cells) and tissues (10 mg). The concentrations of RNA isolated from cells, eight normal colon tissues and 24 colorectal cancer tissues, were measured using a NanoDrop spectrophotometer (Thermo Fisher Scientific). The concentration and size distribution of EV‐RNA isolated from 65 paired small Te‐EVs and 61 paired large Te‐EVs were analysed using a Bioanalyzer (Agilent).

### 2.10 Quantification of Protein Amount

The proteins isolated from 31 paired small Te‐EVs and 36 large Te‐EVs were diluted 10‐fold and measured using a Micro BCA protein assay kit (Thermo Fisher Scientific). The amount of protein isolated from the cells was measured using a TaKaRa BCA Protein Assay Kit (TaKaRa Bio).

### 2.11 Quantitative Analysis of RNA Modification Level

Quantitative analysis of RNA modification levels was performed using Xevo TQ‐XS UHPLC‐UniSpray‐MS/MS (Waters Corporation), as described previously (Monoe et al. 2023, Kogaki et al. 2021). Forty‐two normal and tumour small Te‐EV‐RNA, 48 normal and tumour large Te‐EV‐RNA, eight normal colon tissue RNA, and 24 colorectal cancer tissue RNA samples were subjected to RNA modification analysis. Briefly, 10 ng of the RNA sample was mixed with 1 ng of dG15N5 (internal standard) and treated with nuclease P1 (Wako) at 45°C for 2 h. Thereafter, the ribonucleotides were treated with bacterial alkaline phosphatase (Takara Bio) at 37°C for 2 h. Nucleoside content was measured using a UHPLC‐UniSpray‐MS/MS system. Each nucleoside was quantified from the calibration curve using the ratio of the peak area to that of the internal standard. The ratio of modified nucleosides to nucleosides was calculated based on calibrated concentrations. All peaks were automatically integrated using MASSLYNX XS software (Waters Corporation). Nucleosides were quantified from the calibration curves using the ratio of their peak areas to those of dG15N5 (internal standard).

### 2.12 Chemicals

For readability, the nucleosides are described using abbreviations, and the full names of the nucleosides are listed in Table S2. Compounds A, C, G and I were obtained from Fujifilm Wako Pure Chemical Industries, Ltd. (Tokyo, Japan). S4U was obtained from Abcam (Cambridge, UK), t6A was obtained from BIOLOG (Hayward, California, USA), and m1A, m6A, Im, m1G, ac4C, f5C, mcm5U, m5Um, ca5C, chm5U and Ψ were obtained from Carbosynth, Ltd. (Compton, UK). i6A, D and m5D were obtained from Granlen, Inc. (Zhengzhou, China). m7G and mcm5S2U were obtained from Santa Cruz Biotechnology, Inc. hm5C was obtained from Biosearch Technologies (Hoddesdon, UK), m2G and U were obtained from Sigma‐Aldrich (St. Louis, MO, USA). Am, Gm, m5C, Cm, m5U and Um were obtained from Tokyo Chemical Industry Co., Ltd. (Tokyo, Japan). m6,6A, m6Am, m1I, m2,2G, m3C and S2U were obtained from Toronto Research Chemicals, Inc. (Toronto, Ontario, Canada). dG15N5 was obtained from Cambridge Isotope Laboratories, Inc. (Andover, MA, USA). m6t6A, ncm5U, nchm5U, (R)‐mchm5U, (S)‐mchm5U, mnm5S2U, ncm5S2U, mcm5Um and ncm5Um were synthesised by the Graduate School of Pharmaceutical Sciences at Osaka University (Osaka, Japan). Liquid chromatography‐mass spectrometry‐grade ammonium acetate (CH3COONH4, CAS No. 631‐61‐8) and methanol (MeOH, CAS No. 67‐56‐1) were obtained from Fujifilm Wako Pure Chemical Industries, Ltd.

### 2.13 Immunohistochemistry (IHC)

Tumour tissue samples were placed in Tissue‐Tek OCT Compound (Sakura Finetek), frozen, and cut into 8‐µm‐thick sections. Sections were air‐dried for 30 min and fixed in 10% formalin for 10 min. The sections were then washed thrice with PBS‐T (PBS + 0.02% tween20) and PBS for 3 min each. Sections were permeabilised with 0.2% Triton‐X (Sigma‐Aldrich) for 10 min and washed twice in PBS for 3 min each. Slides were then blocked with peroxidase blocking reagent (BioRad) for 15 min and 4% BSA/PBS for 1 h at 37°C. The sections were stained with anti‐TNF‐α (1:200, ProteinTech), anti‐IL‐6 (1:100, GeneTex), anti‐mouse F4/80 (1:2000, ProteinTech), anti‐Ki67 (1:600, ProteinTech), anti‐CD31 (1:1000, ProteinTech) and anti‐CD68 (1:4000, ProteinTech) antibodies overnight at 4°C. The slides were then washed thrice with PBS‐T and PBS for 3 min each. The sections were incubated in HRP anti‐rabbit IgG secondary antibody (CST) or HRP anti‐goat IgG secondary antibody (1:1000, Thermo Fisher Scientific) and the Envision+ Dual Link System HRP (Agilent) for 30 min at room temperature. The slides were then washed with PBS‐T thrice for 3 min each and developed using a Liquid DAB+ Substrate Chromogen System (Agilent). The slides were counterstained with hematoxylin for 5 min and washed with running water for 3 min. Sections were then dehydrated in 90% EtOH once, 100% EtOH thrice for 1 min each time, and permeabilised with Lemosol (Wako, CAS No. 5989‐27‐5) thrice for 3 min each. The slides were mounted using a MOUNT‐QUICK (DAIDO).

The stained and mounted sections were observed using a research slide scanner VS200 (Olympus). The H‐score was calculated using the HALO software (HALO AI). First, to determine the threshold for positive cells, isotype control‐stained and positive control‐stained tissue samples were trained via machine learning using HALO AI. Positive cells were classified by staining intensity as weak, moderate or strong, and each intensity was assigned 1 point for weak, 2 points for moderate and 3 points for strong. The H‐score was calculated using HALO. Briefly, the H‐score was calculated by multiplying the number of points assigned to each intensity by the percentage of positive cells and then summing them.

### 2.14 PKH26/DiR‐Labelled Te‐EV Incorporation Assay and Cytokine Array

PKH26 1 mM (Sigma‐Aldrich) was diluted to 4 µM in PBS. DiR (Invitrogen) was diluted to 2 µg/mL in PBS. The EV suspension was then mixed in equal volumes with diluted PKH26 (4 µM) or DiR (2 µg/mL) and allowed to react for 5 min. An equal volume of 1% BSA/PBS was added and then centrifuged at 100,000 × *g* for 90 min for SEVs and at 10,000 × *g* for 30 min for LEVs, after which the EVs were recovered. PBMCs (1 × 10^5^ cells) were seeded into 96‐well plates, and PKH26‐labelled Te‐EVs (small Te‐EVs: 3.95 × 10^9^ particles/mL, *n* = 6, large Te‐EVs: 1.7 × 10^8^ particles/mL, *n* = 6) were added. After 24 h, the CM of PBMCs was used for cytokine array analysis. PBMCs were stained with APC‐anti‐CD3 antibody (1:200, Miltenyi), PE‐anti‐CD3 antibody (1:200, Miltenyi), APC‐Vio770 anti‐CD14 antibody (1:200, Miltenyi), VioBlue anti‐CD14 antibody (1:200, Miltenyi), PE‐Vio770 anti‐CD19 antibody (1:200, Miltenyi), and FITC anti‐CD56 antibody (1:100, BioLegend), and then analysed using a ZE5 cell analyzer (BioRad). Cytokine array analysis was performed using the Bio‐Plex Pro Human Cytokine Screening panel, 48‐Plex (BioRad) and Bio‐Plex Pro Human Chemokine Screening panel, 40‐Plex (BioRad), following the manufacturer's protocol. CM diluted four‐fold was used for analysis.

### 2.15 Enzyme‐Linked Immunosorbent Assay (ELISA)

The concentrations of inflammatory cytokines (TNF‐α and IL‐6) in macrophage CM were measured using an ELISA MAX Standard Set (BioLegend). The coating antibody was diluted 200‐fold with coating buffer (pH 9.5) and coated overnight at 4°C on Immuno Clear Standard Modules (Thermo Fisher Scientific). The plate was then washed three times with PBS‐T using a WellwashTM Macroplate Washer (Thermo Fisher Scientific). The samples were diluted 20–200‐fold and reacted with the coated antibodies for 3 h at 37°C. The plates were then washed three times with PBS‐T and incubated with an HRP‐conjugated detection antibody at 37°C for 30 min. Plates were washed four times with PBS‐T, and horseradish peroxidase (HRP) was detected using the TMB Substrate Set (BioLegend). The TMB reaction was stopped with 10% H2SO4, and the absorbance was read at 450 nm using a Multiskan FC microplate reader (Thermo Fisher Scientific).

### 2.16 Cell Proliferation Assay

HT29 (0.5 × 10^3^ cells) and sgAlkbh5 colon26 (0.4 × 10^3^ cells) were seeded into 96‐well plates. The next day, 10 µL of WST‐8 reagent (DOJINDO) was added to the cells and incubated for 2 h, following measurement of OD450 nm‐OD630 nm at Day 0 using a Multiskan FC microplate reader (Thermo Fisher Scientific). The medium was then replaced with a mixture of 75 µL of RPMI adaptive medium and 25 µL of dTHP‐1 CM. The medium was then replaced with a combination of 75 µL RPMI medium and 25 µL dTHP‐1 CM and incubated for the indicated times.

### 2.17 Treatment of dTHP‐1 Cells With Cell Line‐Derived EVs

Cell line‐derived EVs (small EVs: 3.95 × 10^9^ particles/mL, large EVs: 1.70 × 10^8^ particles/mL) were pretreated with or without RNase (10 µg/mL, NIPPON GENE) for 30 min at 37°C and then washed with PBS at 100,000 × *g* for small EVs and 10,000 × *g* for large EVs. CM from dTHP‐1 cells was collected after 48 h of EV treatment. The cells were then centrifuged at 2000 × *g* to remove cell debris and subjected to ELISA.

### 2.18 EV‐RNA Introduction to dTHP‐1 Cells

EV‐RNA (21 ng) was mixed with 2.5 µL of Lipofectamine RNAiMAX (Invitrogen) and introduced into dTHP‐1 cells (1 × 10^5^). dTHP‐1 cell CM was collected 48 h after EV‐RNA treatment. The cells were then centrifuged at 2000 × *g* to remove cell debris and subjected to ELISA.

### 2.19 Demethylation of EV‐RNA by Recombinant ALKBH5 (rALKBH5)

EVs‐RNA (200 ng) was reacted with 400 ng of rALKBH5, 0.3 mM ketoglutaric acid (Wako) and 150 µM FeSO_4_ (Wako) for 4 h at 37°C. Heat‐denatured rALKBH5 was prepared by heating the mixture at 95°C for 10 min. After the reaction, one‐tenth volume of 3 M sodium acetate (Wako) and two volumes of 100% EtOH (Wako) were added and incubated on ice for 30 min. After centrifugation at 10,000 × *g*, the supernatant was discarded. The pellets were washed twice with 70% ethanol, dissolved in water, and used in subsequent experiments.

### 2.20 TLR Inhibition and siRNAs

TLR8 agonist (Motolimod), TLR3 agonist (Poly(I:C)), TLR8 inhibitor (CU‐CPT9a) and TLR3 inhibitor (CU‐CPT‐4a) were purchased from MedChemExpress (Monmonth Junction, NJ, USA). dTHP‐1 cells (1 × 10^5^ cells) were seeded in 24‐well plates and treated with inhibitors for 48 h simultaneously with EVs or agonists.

TLR3 and TLR8 siRNAs were purchased from Thermo Fisher Scientific. The dTHP‐1 cells (1 × 10^5^ cells) were seeded in 24‐well plates and transfected with 20 nM siRNA using 1.0 µL of Lipofectamine RNAiMAX (Thermo Fisher Scientific). Forty‐eight hours after transfection, the culture medium was replaced with a medium containing EVs (small EVs: 3.95 × 10^9^ particles/mL, large EVs: 1.70 × 10^8^ particles/mL). dTHP‐1 cell CM was collected after 48 h of culture, centrifuged at 2000 × *g* to remove cell debris, and subjected to ELISA followed by cell proliferation assays in HT29 or colon26 cells.

### 2.21 RNA‐Sequencing (RNA‐seq) of Te‐EVs

Te‐EV‐RNA was obtained from 5.0 × 10^8^ particles for small Te‐EVs (*n* = 12) or 5.0 × 10^7^ particles for large Te‐EVs (*n* = 12). RNA libraries were constructed using the NEBNext Small RNA Library Prep Set for Illumina (NEB), in accordance with the manufacturer's instructions, and libraries were sequenced on a HiSeq 2500 platform (Illumina) in 75‐base pair single‐end reads. Small RNA‐seq analysis was conducted using the CLC Genomics Workbench v9.5.3 (Qiagen) in accordance with the small RNA alignment and analysis pipeline using default parameters. RNA‐seq data were calculated as the fold‐change between samples using a two‐tailed Student's *t*‐test (*p* < 0.05) in the Subio Platform and Subio Basic Plug‐in (v1.20; Subio, Inc.).

### 2.22 Quantitative PCR (qPCR) for 5′‐half‐GlyGCC

A Mir‐X miRNA First‐Strand Synthesis Kit (Takara, Clontech) was used to prepare cDNA from 10 ng of total EV‐RNA. A CFX Connect 96 plate (BioRad) was used for the qPCR analysis. The following thermal cycling conditions were used: an initial step at 98°C for 30 s, 40 cycles of 95°C for 2 s and 63°C for 5 s for 5′‐half‐GlyGCC and U6. The primer sequences used for gene amplification were as follows: 5′‐half‐GlyGCC forward, 5′‐GGCAGGCGAGAATTCTACCACTGAACCACCAA‐3′ and the mRQ3′ universal primer was used as a reverse primer. The U6 forward and reverse primers were included in the Mir‐X miRNA First‐Strand Synthesis Kit.

### 2.23 5′‐half‐GlyGCC Purification From Te‐EVs

5′‐half‐GlyGCC in EV‐RNA was isolated using Dynabeads M‐270 streptavidin (Thermo Fisher Scientific). Biotinylated 5′‐half‐GlyGCC antisense oligos (50 pmol) were coupled to 10 µL of Dynabeads at room temperature for 10 min. Then, 50 ng of input EV‐RNA was reacted with the oligo‐linked beads for 15 min. Pulled‐down RNA was eluted with 10 µL of water, and 1 µL of RNA was analysed using a Bioanalyzer (Agilent).

### 2.24 In Vitro Transcription of 5′‐half‐tRNAs

In vitro transcription was performed using an RiboMAX Large‐Scale RNA Production System‐T7 (Promega). The transcripts obtained were confirmed using electrophoresis and Sanger sequencing.

### 2.25 Encapsulation of 5′‐half‐GlyGCC and Antisense Into EVs

EV suspension adjusted to 1.0 × 10^9^ particles/100 µL was mixed with one‐tenth volume of 1 M CaCl_2_ and 500 ng of either the 5′‐half‐GlyGCC transcript or its antisense counterpart. The mixture was then allowed to stand at room temperature (18°C–22°C) for 30 min to allow the EV and 5′‐half‐GlyGCC to adhere. EVs were then heat‐shocked at 42°C for 1 min and placed on ice for 5 min. To remove excess 5′‐half‐GlyGCC, EVs were incubated with RNase A (Wako, 10 µg/mL) for 30 min at 37°C. One‐tenth volume of 0.1 M HCl was then added to remove calcium phosphate precipitation. Ultracentrifugation (100,000 × *g*, 90 min) was used to wash the EVs with PBS. The EVs were recovered in PBS and used in subsequent experiments. The antisense sequence was as follows: 5′‐tAcCaCtGaAcCaCcCaTgC‐3′ (Capital: LNA, lower‐case: DNA).

### 2.26 Statistics

Statistical analyses and visualisation quantification were performed using GraphPad Prism software (GraphPad Prism 6.0, GraphPad software). The Mann–Whitney test, Student's *t*‐test, or One‐way ANOVA post hoc Tukey's test was used to assess quantitative data. For between‐group statistical comparisons, the Wilcoxon signed‐rank test was used for paired samples. Statistical significance was set at *p* < 0.05.

## 3 Results

### 3.1 m6A Levels in CRC Tissue‐Exudative EVs (Te‐EVs) Regulate Tumour Progression

**FIGURE 1:**
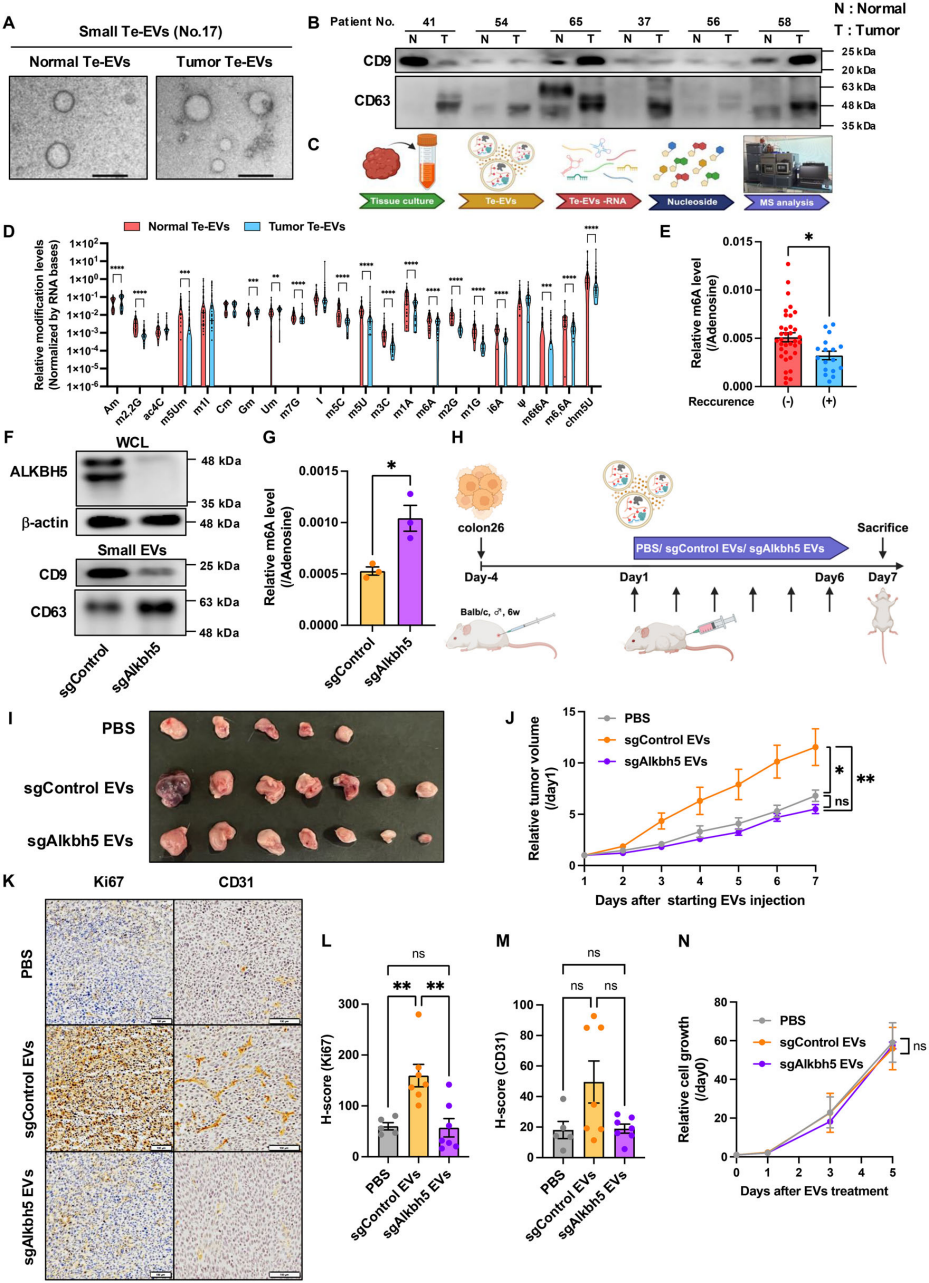
m6A levels in colorectal cancer Te‐EVs regulate tumour progression. (A) Representative images of non‐cancerous colon Te‐EVs (Normal Te‐EVs) and colorectal cancer Te‐EVs (Tumour Te‐EVs). Black bars indicate 200 nm. (B) Western blot analysis of Te‐EVs (Patient No. 37, 41, 54, 56, 58, 65) using an anti‐CD9 and CD63 antibody. (C) Experimental scheme for Te‐EV isolation and subsequent RNA modification analysis. (D) UHPLC‐MS/MS results for normal small Te‐EVs (*n* = 42) and tumour small Te‐EVs (*n* = 42). Wilcoxon signed‐rank test; **p < 0.01, ***p < 0.001, ****p < 0.0001. (E) m6A levels in tumour Te‐EVs from patients with ((+); *n* = 11) or without ((–); *n* = 27) recurrence. Values are presented as the mean ± SEM for each group. Mann–Whitney test; *p < 0.05. (F) Whole‐cell lysates and small EVs obtained from control colon26 cells (sgControl) or Alkbh5 knockout colon26 cells (sgAlkbh5) were subjected to Western blot analysis using anti‐Alkbh5, anti‐β‐actin, anti‐CD9, and anti‐CD63 antibodies. Representative images from three independent experiments are shown. (G) UHPLC‐MS/MS results for sgControl and sgAlkbh5 EVs. Values are presented as the mean ± SEM for each group. Unpaired *t*‐test; *p < 0.05. (H) Experimental scheme for colon26 inoculation and subsequent EV injection. Colon26 cells were injected into BALB/c mice. PBS, sgControl EVs, or sgAlkbh5 EVs were injected i.p., and the tumour size was measured and calculated every day. (I) Representative allograft tumour images. White scale bar, 1 cm. (J) Relative tumour volume. Values are presented as the mean ± SEM for each group. One‐way ANOVA post‐hoc Tukey's test; *p < 0.05, **p < 0.01, ns: not significant. (K) Ki67 and CD31 expression in mouse tumours were examined using immunohistochemical staining. Black bars indicate 100 µm. Representative images of three samples are presented. H‐score for Ki67(L), CD31(M) expression. Values are presented as the mean ± SEM for each group. One‐way ANOVA with Tukey's post hoc test; **p < 0.01, ns: not significant. (N) Colon26 cells were treated with PBS, sgControl EVs, or sgAlkbh5 EVs. Values are presented as the mean ± SEM for each group. One‐way ANOVA with Tukey's post hoc test; ns, not significant. See also Figures S1 and S2.

To characterise the RNA modification profiles in CRC‐derived EVs, we collected normal colon and CRC tissue‐exudative EVs (Te‐EVs) as previously reported (Jingushi et al. 2018), obtaining small and large Te‐EVs (Figures 1A,B and S1A). Resistive pulse sensing (RPS) showed that the number of Te‐EVs did not differ, whereas both small and large CRC‐derived Te‐EVs (tumour Te‐EVs) were significantly larger in diameter than non‐cancerous colon tissue‐derived Te‐EVs (normal Te‐EVs) (Figure S1B–D). Moreover, tumour Te‐EVs contained higher RNA and lower protein levels than normal Te‐EVs (Figure S1E,F). Te‐EV‐derived RNA (Te‐EVs‐RNA) and tissue‐derived RNA were degraded to nucleosides and subjected to semi‐comprehensive RNA modification analysis using UHPLC‐UniSpray‐MS/MS (Figure 1C). We detected 22 RNA modifications in Te‐EVs, 14 of which were downregulated in both small and large tumour Te‐EVs when compared with the levels in normal Te‐EVs (Figures 1D and S2A,B). Meanwhile, only four modifications were downregulated in tumour tissue‐derived RNA compared to those in RNA from adjacent colon tissues (Figure S2C). Differences in most RNA modifications observed in tumour versus normal Te‐EVs were not detected in tissues (Figure S2D). Principal component analysis confirmed distinct RNA modification profiles between normal and tumour Te‐EVs for both small and large EVs (Figure S2E). No significant differences were observed between tumour and normal tissues (Figure S2F). Among the RNA modifications upregulated in tumour Te‐EVs, N6‐methyladenosine (m6A) levels in both small and large Te‐EVs were significantly correlated with patient relapse (Figures 1E and S2G). Moreover, lower m6A levels in tumour Te‐EVs were associated with a poor prognosis (Figure S2H).

m6A demethylase Alkbh5 knockout in the mouse CRC line colon26 verified EVs‐m6A role in tumour progression (Figure 1F). Because the amount of small EVs released per tissue was approximately 40 times higher than that of large CRC‐derived EVs (Figure S1C), experiments were conducted using small EVs. Small EVs from Alkbh5 knockout cells (sgAlkbh5 EVs) showed significantly higher m6A levels than those in control cell‐derived small EVs (sgControl EVs, Figure 1G). Administration of sgControl EVs, but not sgAlkbh5 EVs, promoted colon26 tumour progression in mice (Figure 1H‐J). sgAlkbh5 EVs had no significant effect on the expression of proliferation marker Ki67, which was increased by sgControl EV administration (Figure 1K,L). sgControl EV administration tended to increase the expression of angiogenesis marker CD31, but this effect was attenuated by sgAlkbh5 EVs treatment (Figure 1K,M). Furthermore, neither sgControl EVs nor sgAlkbh5 EVs affected colon26 cell proliferation in vitro (Figure 1N), suggesting that the decrease in m6A levels in cancer EVs promotes tumour progression through stromal cells within the tumour microenvironment rather than directly influencing cancer cells.

### 3.2 Te‐EV m6A Levels Promote Macrophage Inflammation and Cancer Cell Proliferation

**FIGURE 2:**
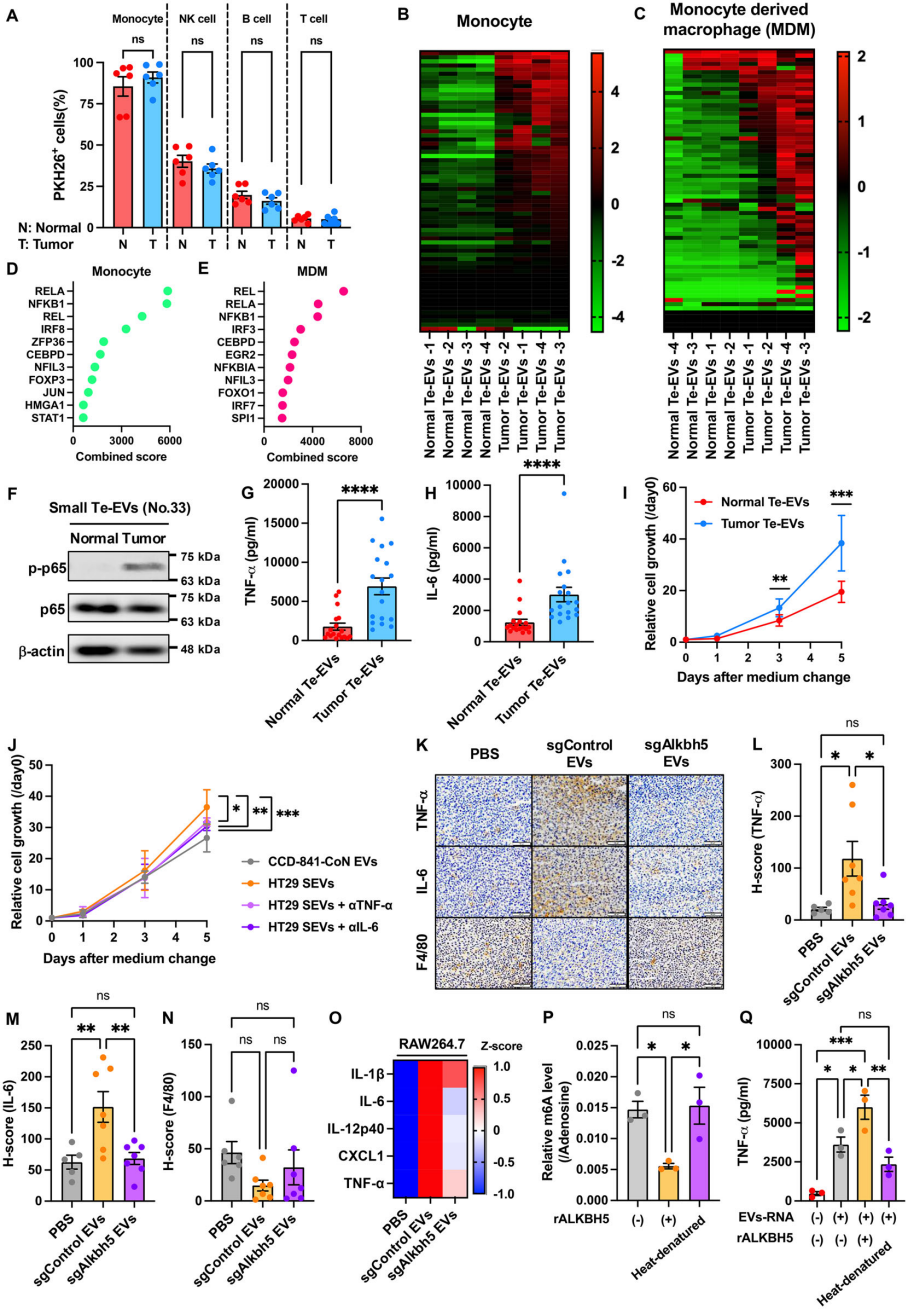
Colorectal cancer‐small Te‐EVs promote inflammation in macrophages and cancer cell proliferation. (A) Normal colon small EVs (N) and colon cancer tissue small EVs (T) were labelled with PKH26 and added to PBMCs. The percentage of PKH26‐positive EVs in each PBMC sample was measured using flow cytometry. Values are presented as the mean ± SEM for each group. Wilcoxon signed‐rank test; ns, not significant. (B) Heatmap of the cytokine array performed using conditioned medium from monocytes (B) or monocyte‐derived macrophages (MDM, C) treated with normal (*n* = 4) or tumour (*n* = 4) small Te‐EVs. Enrichment analysis of the cytokine array using conditioned medium from monocytes (D) or MDM (E) treated with normal (*n* = 4) or tumour (*n* = 4) small Te‐EVs. (F) Whole‐cell lysates obtained from normal or tumour small Te‐EV‐treated MDM were subjected to Western blot analysis using anti‐phospho p65 antibody, anti‐p65 antibody and anti‐β‐actin antibody. Representative images of three independent experiments are shown. ELISA was conducted using conditioned medium from MDM treated with normal (*n* = 18) or tumour (*n* = 18) small Te‐EVs. TNF‐α (G) and IL‐6 (H) levels. Values are presented as the mean ± SEM for each group. Wilcoxon signed‐rank test; ****p < 0.0001. (I) HT29 cells were cultured in a conditioned medium from MDM treated with normal (*n* = 18) or tumour (*n* = 18) small Te‐EVs. Values are presented as the mean ± SEM for each group. Wilcoxon signed‐rank test: **p < 0.01, ***p < 0.001. (J) HT29 cells were cultured in a conditioned medium with differentiated THP‐1 (dTHP‐1) cells treated with CCD‐841‐CoN small EVs or HT29 small EVs alone or in combination with TNF‐α or IL6 antibodies. Values are presented as the mean ± SEM for each group. One‐way ANOVA post hoc Tukey's test; *p < 0.05, **p < 0.01, ***p < 0.001. (K) Immunohistochemical analysis of TNF‐α, IL‐6 and F4/80 expression in the tumour tissues of mice inoculated with small EVs obtained from Alkbh5 knockout (sgAlkbh5 EVs) or control colon26 cells (sgControl EVs). Scale bar: 100 µm. Representative images of five (PBS group) and seven (sgControl EVs and sgAlkbh5 group) independent experiments are shown. H‐scores of TNF‐α (L), IL‐6 (M) and F4/80 (N) expression. Values are presented as the mean ± SEM for each group. One‐way ANOVA post hoc Tukey's test; *p < 0.05. ns, not significant. (O) Heatmap of cytokine array using conditioned medium from RAW264.7 cells treated with sgControl EVs (*n* = 4) or sgAlkbh5 EVs (*n* = 4). (P) HT29‐derived EV‐RNA was reacted with recombinant ALKBH5 (rALKBH5), and m6A levels were measured. Heat‐denatured rALKBH5 served as a negative control. Values are presented as the mean ± SEM for each group. One‐way ANOVA post hoc Tukey's test; *p < 0.05, ns, not significant. (Q) HT29‐derived EV‐RNA was pretreated with or without rALKBH5 and introduced into dTHP‐1 cells. TNF‐α concentration was then measured via ELISA using conditioned medium from dTHP‐1 cells. Heat‐denatured rALKBH5 served as a negative control. Values are presented as the mean ± SEM for each group. One‐way ANOVA post hoc Tukey's test; *p < 0.05, **p < 0.01, ***p < 0.001. ns, not significant. See also Figures S3–S6.

A previous study suggested that the tumour immune microenvironment is closely associated with CRC development, and we then focused on the effects on immune cells. Peripheral blood mononuclear cells (PBMCs) were treated with Te‐EVs, and EV uptake was analysed using flow cytometry (Figure S3A,B). Regardless of EV origin or EV staining dye, monocytes exhibited the highest uptake of small and large EVs (Figures 2A, S3C,D and S4A). Therefore, we performed cytokine arrays on monocytes treated with small and large Te‐EVs. Thirty‐five cytokines and 32 cytokines were upregulated in tumour versus normal Te‐EV‐treated monocytes by small EVs and large EVs, respectively(Figures 2B S4B and Table S3). Enrichment analysis showed that NF‐kB‐related factors (REL, RELA and NFKB1) were enriched in tumour Te‐EVs (Figures 2D and S4D). Most infiltrating monocytes differentiate into macrophages in the tumour microenvironment (Long et al. 2019). Primary human macrophages were used to verify the effects of the tumour Te‐EVs. Twenty‐six and nineteen cytokines were upregulated in macrophages treated with small and large tumour Te‐EVs, respectively, compared to normal Te‐EV‐treated macrophages (Figures 2C S4C and Table S4). Enrichment analysis confirmed the upregulation of REL, RELA and NFKB1 (Figures 2E and S4E). Tumour Te‐EVs upregulated the phosphorylation levels of NF‐kB subunit p65 (Figures 2F and S4F), promoting downstream TNF‐α and IL‐6 secretion (Figures 2G,H and S4G,H) in both small and large tumour Te‐EV‐treated macrophages. CM from both small and large tumour Te‐EV‐treated macrophages significantly promoted the proliferation of HT29 cells (Figures 2I and S4I).

As tumour Te‐EVs include stromal cell‐derived EVs, we sought to corroborate our findings using cancer cell line‐derived EVs. We collected small and large EVs from the normal human colon epithelial cell line CCD‐841‐CoN and human CRC cell lines HT29 and SW620, which were confirmed by transmission electron microscopy (TEM) and nanoparticle analysis (Figure S5A,B). Human THP‐1 monocytes were differentiated into macrophage‐like cells (dTHP‐1 cells). As for tumour Te‐EVs, small and large EVs from both HT29 and SW620 cells, but not CDD‐841‐CoN cells, significantly promoted TNF‐α and IL‐6 secretion by dTHP‐1 cells (Figure S5C,D). CM obtained from HT29‐derived small EV‐treated dTHP‐1 cells promoted HT29 cell proliferation, but its effects were neutralised by treatment with anti‐TNF‐α or anti‐IL‐6 antibodies (Figure 2J). These results suggested that CRC‐derived EVs promote tumorigenesis by activating the NF‐kB pathway to stimulate TNF‐α and IL‐6 secretion by macrophages.

IHC analysis showed that TNF‐α and IL‐6 expression was significantly upregulated in the sgControl EV‐treated group, while this effect was attenuated in the sgAlkbh5 EV group (Figure 2K‐M). No significant differences in F4/80‐positive macrophage abundance were noted between any of the groups (Figure 2K,N). Cytokine arrays revealed enhanced secretion of NF‐kB pathway‐associated cytokines in RAW264.7 macrophages treated with sgControl EVs (Figure 2O and Table S3). This upregulation was not observed in sgAlkbh5 EVs relative to that in PBS‐treated controls (Figure 2O and Table S5). Furthermore, sgAlkhb5 HT29 cell‐derived small EVs also did not increase TNF‐α and IL‐6 secretion by dTHP‐1 cells (Figure S6).

Based on these results, we hypothesised that EVs‐RNA m6A levels contribute to macrophage‐driven inflammation. We confirmed enhanced TNF‐α and IL‐6 secretion by dTHP‐1 cells treated with CRC cell line‐derived EVs‐RNA (Figure S5E,F). This increase occurred even after cell‐free RNAs were degraded by RNase or removed via size exclusion chromatography (SEC) prior to uptake (Figure S5G‐I), suggesting that CRC‐derived EVs‐RNA triggered inflammatory responses in macrophages. EVs‐RNA m6A levels were reduced by wild‐type recombinant ALKBH5 protein (rALKBH5), as was TNF‐α secretion by dTHP‐1 cells treated with m6A‐depleted EVs‐RNA. Heat‐denatured rALKBH5 failed to decrease EV‐RNA m6A levels and the enhanced TNF‐α secretion in dTHP‐1 cells (Figure 2P,Q). Collectively, these results suggest that the reduction of m6A levels on EVs‐RNA promotes inflammatory cytokine secretion by macrophages.

### 3.3 Association of m6A Levels in Tumour Te‐EVs With Inflammatory Status of Clinical Tumour Tissues

**FIGURE 3:**
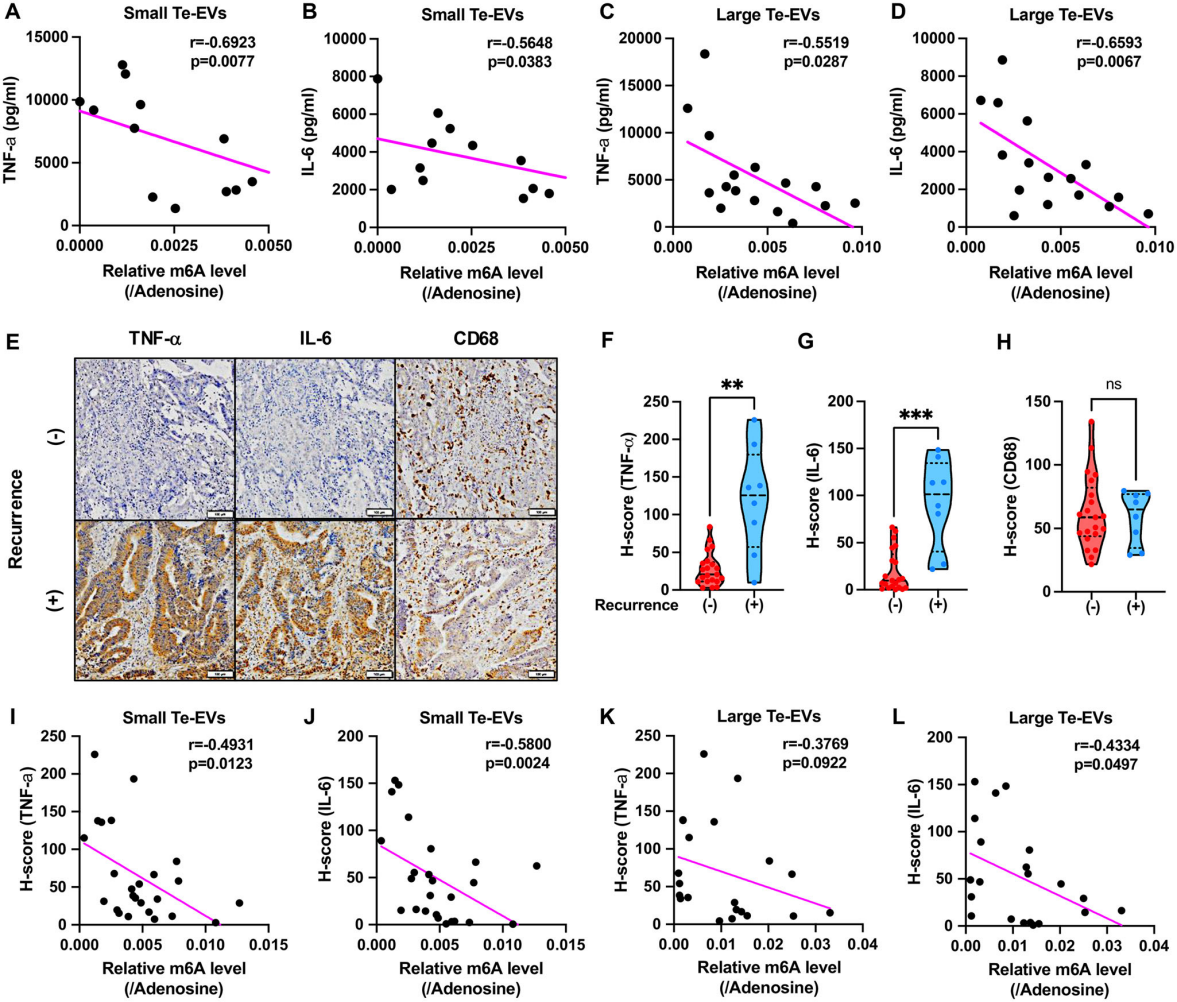
Association of m6A levels in tumour Te‐EVs with inflammatory status of clinical tumour tissues. Correlation analysis of m6A levels in tumour small Te‐EVs and the amount of TNF‐α (A) or IL‐6 (B) released from tumour small Te‐EV‐treated monocyte‐derived macrophages (MDMs). Spearman's correlation coefficient *r* was calculated. Correlation analysis of m6A levels in tumour large Te‐EVs and the amount of TNF‐α (C) or IL‐6 (D) released from tumour large Te‐EV‐treated MDMs. Spearman's correlation coefficient *r* was calculated. (E) Immunohistochemical analysis of TNF‐α, IL‐6 and CD68 expression in colorectal cancer clinical tissue with or without recurrence. Representative images are shown (21 tumour tissues obtained from recurrence‐free patients and eight tumour tissues from relapsed patients). The scale bar shows 100 µm. H‐score of TNF‐α (F) IL‐6 (G) and CD68 (H) expression. Mann–Whitney test; **p < 0.01, ***p < 0.001, ns, not significant. Correlation analysis of m6A levels in tumour small Te‐EVs and the H‐scores of TNF‐α (I) or IL‐6 (J) in corresponding colorectal cancer clinical tissues. Spearman's correlation coefficient (*r*) was calculated. Correlation analysis of m6A levels in tumour large Te‐EVs and the H‐scores of TNF‐α (K) or IL‐6 (L) in corresponding colorectal cancer clinical tissues. Spearman's correlation coefficient (*r*) was calculated.

We examined the correlation between tumour Te‐EV m6A levels and TNF‐α and IL‐6 secretion by tumour Te‐EV‐treated dTHP‐1 cells. TNF‐α and IL‐6 secretion was negatively correlated with m6A levels in both small and large tumour Te‐EVs (Figure 3A‐D). IHC and tumour Te‐EV recovery revealed that TNF‐α and IL‐6 were significantly upregulated in CRC tissues from patients with recurrent disease compared with tissues from patients without recurrence, while the abundance of CD68‐positive macrophages was not different (Figure 3E‐H). Furthermore, m6A levels in both large and small Te‐EVs were negatively correlated with TNF‐α and IL‐6 levels in CRC tissues (Figure 3I‐L).

### 3.4 Tumour EV m6A Levels Regulate Macrophage Inflammatory Responses via TLR8

**FIGURE 4:**
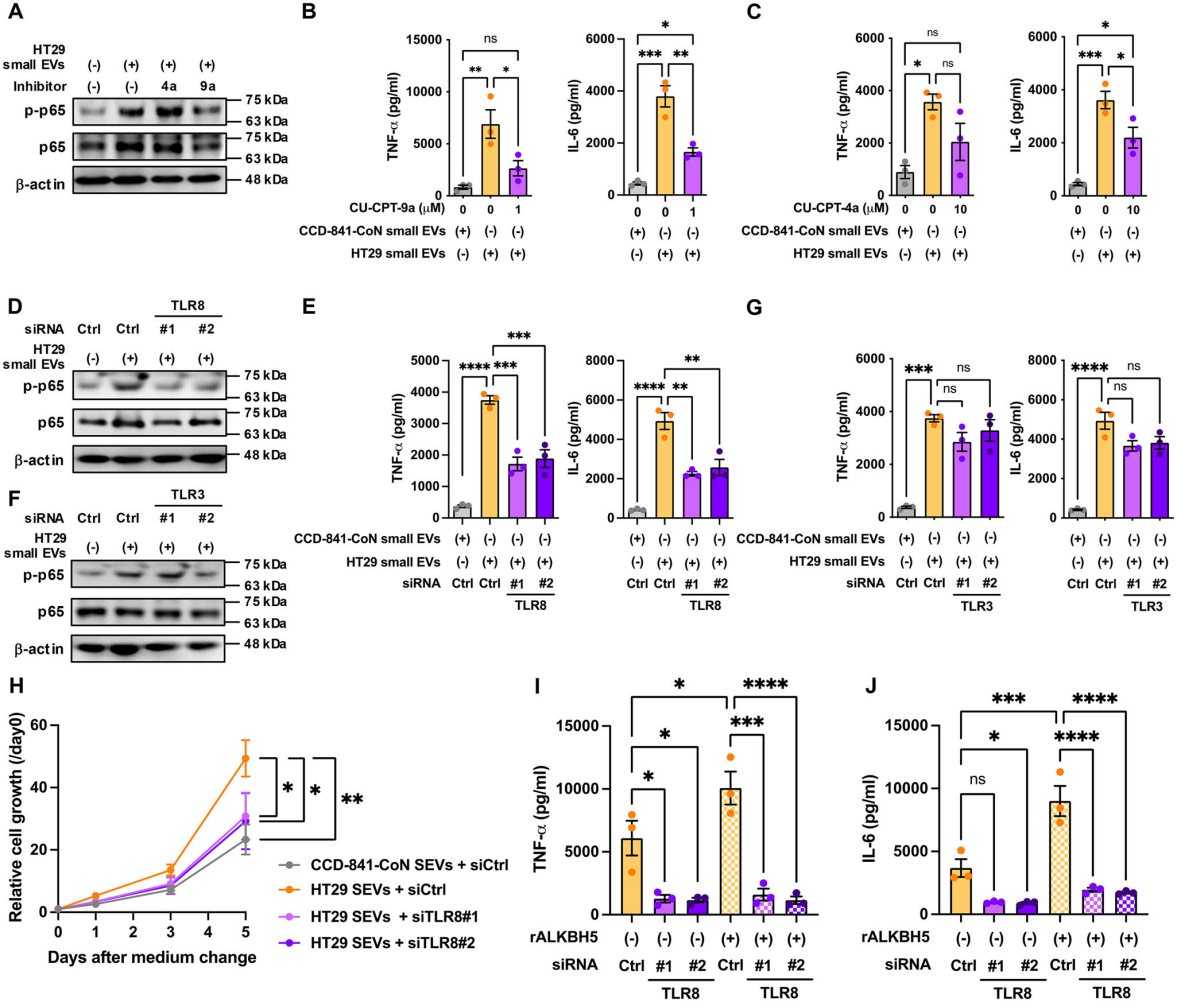
m6A levels in EVs regulate inflammatory responses via TLR8 in macrophages. Differentiated THP‐1 cells (dTHP‐1) were pretreated with or without CU‐CPT‐9a and CU‐CPT‐4a. (A) Whole‐cell lysates obtained from dTHP‐1 cells were subjected to Western blot analysis using an anti‐phospho p65 antibody, anti‐p65 antibody and anti‐β‐actin antibody. Representative images from three independent experiments are shown. Conditioned medium from dTHP‐1 cells treated with CCD‐841‐CoN or HT29 small EVs was used for ELISA. TNF‐α (B, left and C, left), and IL‐6 (B, right and C, right). Values are presented as the mean ± SEM for each group. One‐way ANOVA post hoc Tukey's test; *p < 0.05, **p < 0.01, ***p < 0.001. ns, not significant. dTHP‐1 cells were pre‐transfected with or without TLR8 siRNA. (D) Whole‐cell lysates obtained from dTHP‐1 cells treated with CCD‐841‐CoN small EVs or HT29 small EVs were subjected to Western blot analysis using an anti‐phospho p65 antibody, anti‐p65 antibody and anti‐β‐actin antibody. Representative images from three independent experiments are shown. Conditioned medium was used for the ELISA. TNF‐α (E, left) and IL‐6 (E, right). Values are presented as the mean ± SEM for each group. One‐way ANOVA post hoc Tukey's test; **p < 0.01, ***p < 0.001, ****p < 0.0001. dTHP‐1 cells were pre‐transfected with or without TLR3 siRNA. (F) Whole‐cell lysates obtained from dTHP‐1 cells treated with CCD‐841‐CoN small EVs or HT29 small EVs were subjected to Western blot analysis using an anti‐phospho p65 antibody, anti‐p65 antibody and anti‐β‐actin antibody. Representative images from three independent experiments are shown. Conditioned medium was used for ELISA. TNF‐α (G, left) and IL‐6 (G, right). Values are presented as the mean ± SEM for each group. One‐way ANOVA post hoc Tukey's test; ***p < 0.001, ****p < 0.0001; ns, not significant. (H) HT29 cells were cultured in a conditioned medium from dTHP‐1 cells transfected with or without TLR8 siRNA and CCD‐841‐CoN small EVs or HT29 small EVs. Values are presented as the mean ± SEM for each group. One‐way ANOVA post hoc Tukey's test; *p < 0.05, **p < 0.01. HT29‐derived EVs‐RNA were pretreated with or without rALKBH5 and introduced into dTHP‐1 cells transfected with or without TLR8 siRNA. TNF‐α (I) and IL‐6 (J) concentrations were measured via ELISA using conditioned medium from dTHP‐1 cells. Values are presented as the mean ± SEM for each group. One‐way ANOVA post hoc Tukey's test; *p < 0.05, ***p < 0.001, ****p < 0.0001, ns, not significant. See also Figure S7.

Treatment of dTHP‐1 cells with TLR inhibitors and HT29 cell‐derived EVs revealed that the TLR8 inhibitor, CU‐CPT‐9a, significantly suppressed the upregulation of p65 phosphorylation levels elicited by both small HT29 and SW620 EVs (Figures 4A and S7A). TNF‐α and IL‐6 secretion were promoted by small and large HT29 EVs, and small SW620 EVs also suppressed by CU‐CPT‐9a treatment (Figures 4B and S7B,D). In contrast, the TLR3 inhibitor CU‐CPT‐4a failed to produce a comparable effect (Figures 4A,C and S7A,C,E). To further investigate TLR involvement, we silenced TLR8 and TLR3 in dTHP‐1 cells using siRNAs (Figure S7F,G). TLR8 knockdown attenuated the effects of small and large HT29 EVs and small SW620 EVs on p65 phosphorylation levels, TNF‐α and IL6 secretion by dTHP‐1 cells (Figures 4D,E and S7H,J), whereas TLR3 knockdown had no significant attenuating effect (Figures 4F,G and S7I,K). In addition, CM obtained from TLR8 knockdown dTHP‐1 cells treated with either small or large HT29 EVs failed to promote HT29 cell proliferation (Figures 4H and S7L). The upregulation of TNF‐α and IL‐6 secretion induced by rALKBH5‐treated EVs‐RNA was significantly repressed by TLR8 knockdown in dTHP‐1 cells (Figure 4I,J), suggesting that m6A levels in EV‐RNA trigger an inflammatory response via TLR8 in macrophages, thereby promoting tumorigenesis.

### 3.5 5’‐half‐GlyGCC Is Enriched in Both Small and Large Tumour Te‐EVs With Reduced m6A Levels

**FIGURE 5:**
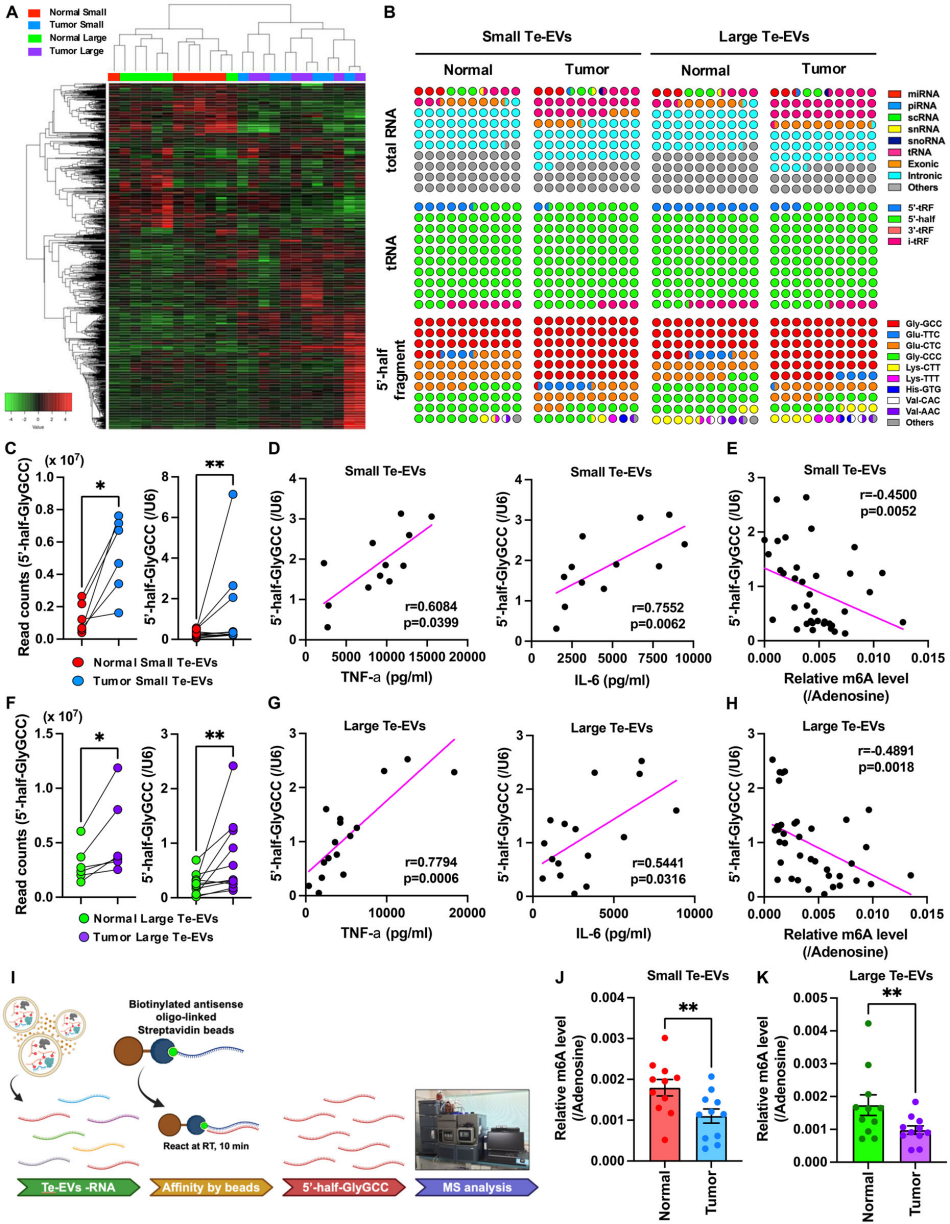
5′‐half‐GlyGCC is enriched in both small and large tumour Te‐EVs with reduced m6A levels. (A) Heatmap of small RNA‐seq data obtained from normal (*n* = 6) and tumour (*n* = 6) small and large Te‐EVs. (B) Percentage dot plot of each RNA species, tRNA fragment species, and tRF‐encoded amino acid species in Te‐EVs from small RNA‐seq data. Read counts of 5′‐half‐GlyGCC in normal (*n* = 6) and tumour (*n* = 6) small Te‐EVs (C, left). Quantitative PCR results for 5′‐half‐GlyGCC (C, right, *n* = 11). Correlation analysis of 5′‐half‐GlyGCC levels in small Te‐EVs and the amount of TNF‐α (D, left) or IL‐6 (D, right) released from small Te‐EV‐treated monocyte‐derived macrophage (MDMs). Spearman's correlation coefficient (*r*) was calculated. (E) Correlation analysis of m6A and 5′‐half‐GlyGCC levels in small Te‐EVs. Spearman's correlation coefficient (*r*) was calculated. Read counts of 5′‐half‐GlyGCC in normal (*n* = 6) and tumour large (*n* = 6) Te‐EVs (F, left). Quantitative PCR results for 5′‐half‐GlyGCC (F, right, *n* = 11). Correlation analysis of 5′‐half‐GlyGCC levels in large Te‐EVs and the amount of TNF‐α (G, left) or IL‐6 (G, right) released from large Te‐EV‐treated MDMs. Spearman's correlation coefficient (*r*) was calculated. (H) Correlation analysis of m6A and 5′‐half‐GlyGCC levels in large Te‐EVs. Spearman's correlation coefficient (*r*) was calculated. (I) Experimental scheme for Te‐EVs: RNA isolation, pull‐down of 5′‐half‐GlyGCC, and subsequent RNA modification analysis. m6A levels of 5′‐half‐GlyGCC obtained from normal and tumour‐derived EV‐RNA. Small (J) and large Te‐EVs (K). Values are presented as the mean ± SEM for each group. Wilcoxon signed‐rank test; **p < 0.01 (*n* = 11). See also Figures S8 and S9.

To identify RNA molecules that act as ligands for TLR8, we performed small RNA‐seq of Te‐EVs. We found that tumour Te‐EVs had characteristic RNA profiles compared to those of normal Te‐EVs (Figure 5A). Moreover, normal small and large Te‐EVs had distinct compositions, whereas tumour small and large Te‐EVs had similar profiles (Figure 5A, Table S6). When the detected transcripts were classified by RNA species, tRNA was enriched in both large and small tumour Te‐EVs compared with normal Te‐EVs (Figures 5B and S8A). We also found that most of these RNA molecules were tRNA fragments, especially 5′‐tRNA halves (5′‐half), and that more than half of the 5′‐half sequences were glycine‐GCC (GlyGCC, Figure 5B). RNA‐seq and qPCR analyses showed that 5′‐half‐GlyGCC was significantly enriched in both small and large tumour Te‐EVs compared to that in normal Te‐EVs (Figure 5C,F). 5′‐half‐GluTTC, 5′‐half‐GluCTC and 5′‐half‐GlyCCC were also abundant in Te‐EVs; however, there was no significant difference between normal and tumour Te‐EVs (Figure S8B,C). TNF‐α and IL‐6 secretion levels correlated with 5′‐half‐GlyGCC levels in both small and large Te‐EVs (Figure 5D,G). Moreover, 5′‐half‐GlyGCC levels negatively correlated with m6A levels in Te‐EVs from both small and large Te‐EVs (Figure 5E,H). To determine whether m6A on 5′‐half‐GlyGCC is reduced in tumour Te‐EVs compared with normal Te‐EVs, we isolated 5′‐half‐GlyGCC from Te‐EVs and analysed the associated m6A levels (Figures 5I and S9). MS analysis showed that m6A levels were significantly decreased in both small and large tumour Te‐EVs compared to those in normal Te‐EVs (Figure 5J,K).

### 3.6 5′‐half‐GlyGCC in Tumour EVs Promotes Tumour Growth In Vivo

**FIGURE 6:**
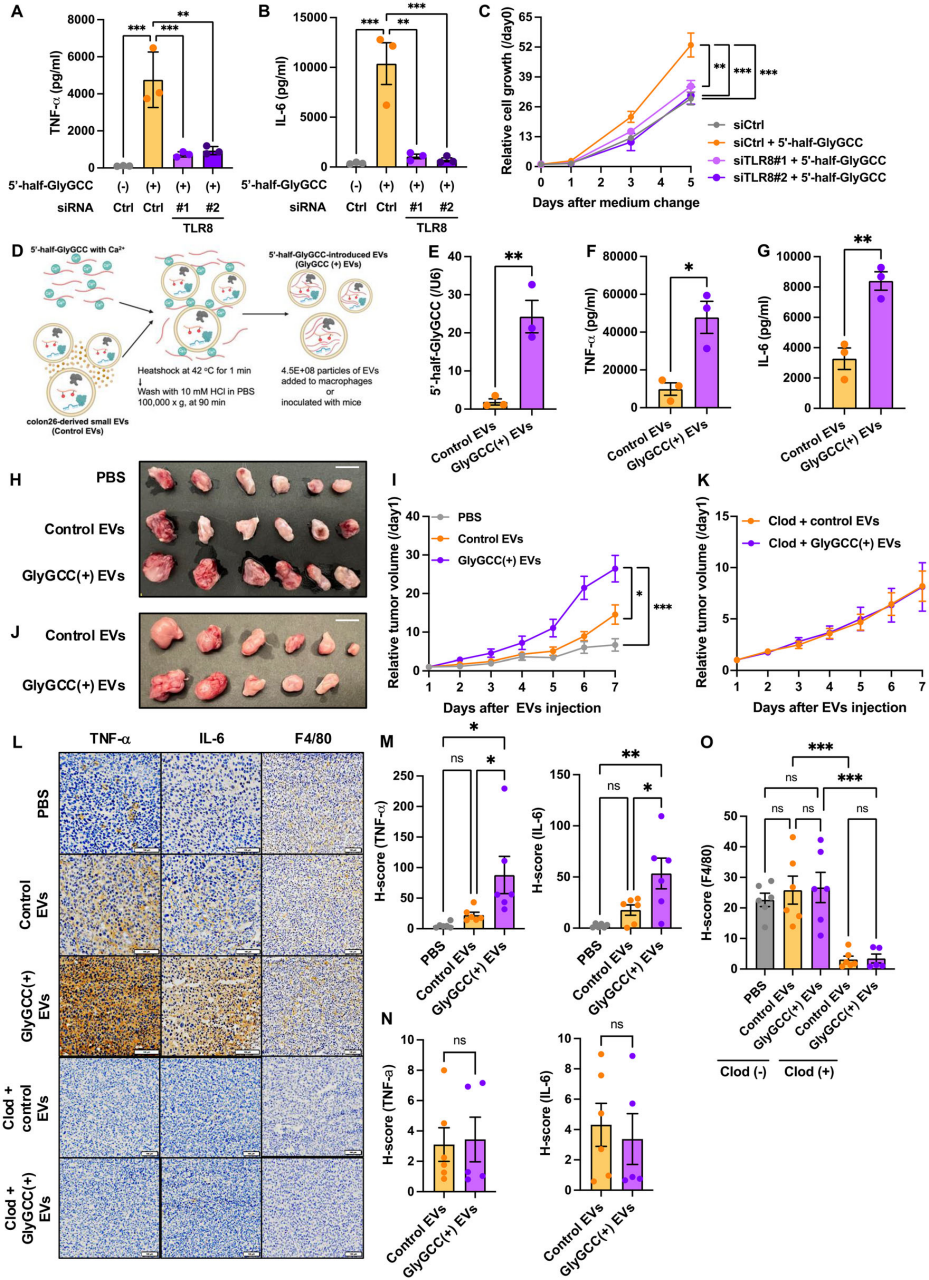
5′‐half‐GlyGCC in tumour EVs promotes tumour growth in vivo. Differentiated THP‐1 cells (dTHP‐1) were pre‐transfected with or without TLR8 siRNA and 5′‐half‐GlyGCC. Conditioned medium from dTHP‐1 cells was used for the ELISA. TNF‐α (A) and IL‐6 (B). Values are presented as the mean ± SEM for each group. One‐way ANOVA post hoc Tukey's test; **p < 0.01, ***p < 0.001. (C) HT29 cells were cultured in a conditioned medium from dTHP‐1 cells transfected with or without TLR8 siRNA and 5′‐half‐GlyGCC. Values are presented as the mean ± SEM for each group. One‐way ANOVA post hoc Tukey's test; **p < 0.01, ***p < 0.001. (D) Experimental scheme for the introduction of 5′‐half‐GlyGCC into colon26 small EVs and subsequent analysis. (E) Results of quantitative PCR of 5′‐half‐GlyGCC‐loaded colon26 small EVs (GlyGCC(+) EVs). Values are presented as the mean ± SEM for each group. Unpaired *t*‐test; **p < 0.01. ELISA was conducted using conditioned medium from RAW264.7 cells treated with GlyGCC(+) EVs. TNF‐α (F) and IL‐6 (G) concentration. Values are presented as the mean ± SEM for each group. Unpaired *t*‐test; *p < 0.05, **p < 0.01. Colon26 cells were injected into BALB/c mice. PBS, Control EVs or GlyGCC(+) EVs were injected i.p. Representative xenograft tumour images (H) are shown. White scale bar, 1 cm. Tumour size was measured and calculated daily (I). Values are presented as the mean ± SEM for each group. One‐way ANOVA post hoc Tukey's test; *p < 0.05, ***p < 0.001. Colon26 cells were injected into BALB/c mice. PBS, Control EVs or GlyGCC(+) EVs and clodronate liposomes were injected i.p. Representative xenograft tumour images (J) are shown. White scale bar, 1 cm. Tumour size was measured and calculated every day (K). Values are presented as the mean ± SEM for each group. Unpaired *t*‐test; ns, not significant. (L) Immunohistochemical analysis of TNF‐α, IL‐6 and F4/80 expression in tumour tissues of mice inoculated with small EVs obtained from GlyGCC(+) EVs and clodronate liposomes. Scale bar: 100 µm. Representative images of six (PBS, Control EVs, GlyGCC(+) EVs, and Control EVs with clodronate liposomes) or five (GlyGCC(+) EVs with clodronate liposomes) independent experiments are shown. H‐scores of TNF‐α (M, left) and IL‐6 (M, right) expression in GlyGCC(+) EV injection experiments. Values are presented as the mean ± SEM for each group. One‐way ANOVA post hoc Tukey's test; *p < 0.05, **p < 0.01, ns, not significant. H‐score of TNF‐α (N, left) and IL‐6 (N, right) expression in GlyGCC(+) EVs combined with clodronate liposome injection. Values are presented as the mean ± SEM for each group. Unpaired *t*‐test; ns, not significant. (O) H‐score of F4/80 expression in GlyGCC(+) EVs, with or without clodronate liposome injection. Values are presented as the mean ± SEM for each group. Unpaired *t*‐test; ns, not significant. See also Figures S10–S12.

To verify whether 5′‐half‐GlyGCC in tumour EVs promotes tumorigenesis, we prepared unmodified 5′‐half‐GlyGCC via in vitro transcription. The introduction of 5′‐half‐GlyGCC into dTHP‐1 cells induced the secretion of TNF‐α and IL‐6, which was impeded by TLR8 knockdown (Figure 6A,B). In contrast, the introduction of 5′‐half‐GluTTC, 5′‐half‐GluCTC or 5′‐half‐GlyCCC into dTHP‐1 cells had no significant effect on TNF‐α (Figure S10A) or IL‐6 (Figure S10B) secretion. CM obtained from 5′‐half‐GlyGCC‐treated dTHP‐1 cells significantly upregulated HT29 cell proliferation, and this effect was impeded by TLR8 knockdown in dTHP‐1 cells (Figure 6C). Next, we attempted to encapsulate 5′‐half‐GlyGCC into colon26‐derived small EVs (GlyGCC (+) EVs; Figures 6D and S11A,B). Subsequently, excess 5′‐half‐GlyGCC was digested via RNase treatment. The 5′‐half‐GlyGCC levels were elevated in GlyGCC (+) EVs compared to control EVs (Figure 6E). We also examined the ability of GlyGCC (+) EVs to induce the secretion of inflammatory cytokines by macrophages. GlyGCC (+) EVs significantly upregulated TNF‐α and IL‐6 secretion relative to that induced by control colon26 small EVs (Control EVs) in RAW264.7 macrophages (Figure 6F,G).

GlyGCC (+) EVs significantly upregulated colon26 tumour growth compared to PBS and control EV treatments (Figure 6H,I). To verify whether GlyGCC (+) EV‐induced upregulation of tumour growth was mediated by macrophages, clodronate liposomes were used. Clodronate liposomes negated the effect of GlyGCC (+) EVs on tumour growth (Figure 6J,K). IHC analysis of TNF‐α and IL‐6 in tumour tissues showed that GlyGCC (+) EVs increased TNF‐α and IL‐6 expression compared to control EV treatment (Figure 6L,M). Furthermore, clodronate liposomes impeded the effect of GlyGCC (+) EVs on TNF‐α and IL‐6 expression and decreased F4/80 expression (Figure 6L,N,O).

Finally, we examined the tumour‐promoting potential of 5′‐half‐GlyGCC using normal and tumour Te‐EVs. Normal Te‐EVs encapsulated with 5′‐half‐GlyGCC upregulated p65 phosphorylation levels and subsequently increased TNF‐α and IL‐6 secretion via TLR8 in dTHP‐1 cells (Figure S12A,B). Conversely, introduction an antisense targeting 5′‐half‐GlyGCC to tumour Te‐EVs abrogated p65 phosphorylation and reduced TNF‐α and IL‐6 secretion in dTHP‐1 cells via TLR8 (Figure S12C,D). These results highlight the tumour‐promoting potential of 5′‐half‐GlyGCC in tumour EVs.

## 4 Discussion

In this study, we demonstrate that cancer cells utilise RNA modification in EVs, a mechanism typically used to protect against viral and bacterial invasion. We found that cancer‐specific modified RNAs encapsulated in tumour‐derived EVs promote tumorigenesis via macrophages in the tumour microenvironment. Small EVs mainly include exosomes, while large EVs include microvesicles and carry distinct molecular cargo (Mariscal et al. 2020, Luo et al. 2020). However, no comparative studies have reported on nucleic acid and RNA modifications in EVs. Small RNA‐seq analysis of normal colon Te‐EVs revealed distinct small RNA profiles between small and large Te‐EVs. These profiles were similar between small and large tumour Te‐EVs, which also shared a similar tumour‐promoting potential. This suggests that dysregulated RNA loading into EVs in CRC may favour the encapsulation of immune‐stimulating RNA, leading to tumour progression.

Our RNA modification analysis identified 22 modifications in EVs, many of which were downregulated in tumour Te‐EVs. Among these, m5C, N7‐methylguanosine (m7G) and N1‐methyladenosine (m1A) regulate TLR responsiveness (Cui et al. 2022), indicating that decreased EV‐RNA modifications may induce immune responses.

Co‐culture of CRC cells with THP‐1 cells increases 5′‐half‐GlyGCC expression in THP‐1 cells (Wu et al. 2021). Moreover, 5′‐half‐GlyGCC is more abundant in the plasma of CRC patients than in that of healthy controls (Wu et al. 2021), supporting our findings that 5′‐half‐GlyGCC is encapsulated in CRC cell‐derived EVs. ALKBH3, upregulated in CRC cells, is responsible for 5′‐half‐GlyGCC generation (Chen et al. 2019). In breast cancer cells, 5′‐half‐GlyGCC binds to FTO, an m6A eraser, stimulating FTO demethylase activity (Chen et al. 2023), suggesting that high expression of 5′‐half‐GlyGCC leads to decreased m6A levels. Furthermore, METTL14, an m6A writer, is significantly lower in CRC tissues than in normal colon tissues, correlating with poor CRC prognosis (Yang et al. 2020). Thus, CRC EVs contain high levels of 5′‐half‐GlyGCC with low m6A levels, contributing to CRC progression.

Patients with CRC tumours exhibiting high IL‐6 and TNF‐α expression have lower disease‐free survival (DFS) (Toyoshima et al. 2019, Grimm et al. 2010). Patients with high blood IL‐6 levels also have lower DFS (Shiga et al. 2016, Cheng et al. 2023). IL‐6 plays a pivotal role in acquiring cancer stem cell properties via FRA1 transcription factor activation in colon cancer cells (Wang et al. 2019). The number of Treg infiltrates in colorectal cancer tissues correlates with DFS (Saito et al. 2016). Higher TNF‐α expression in colon cancer tissues leads to increased macrophage infiltrates expressing CCL18+, the ligand for CCR8, along with more CCR8+ Treg infiltrates (Guo et al. 2023). Although the number of tumour‐infiltrating macrophages was not associated with disease recurrence, TNF‐α and IL‐6 were highly expressed in the recurrence group. Additionally, m6A levels in colorectal cancer Te‐EVs correlated with TNF‐α and IL‐6 levels in the paired cancer tissue, suggesting that while colorectal cancer EVs do not affect the number of tumour‐infiltrating macrophages, they may contribute to recurrence by increasing the production of TNF‐α and IL‐6 production in macrophages. Therefore, reduced m6A levels in tumour‐derived EVs may promote CRC recurrence by enhancing IL‐6 and TNF‐α production by macrophages within the tumour.

We identified 5′‐half‐GlyGCC as an effector sequence. Kirino et al. reported that the tRNA Val half acts on TLR7 in macrophages, leading to an increase in the expression of inflammatory cytokines such as TNF‐α. In our data, the tRNA Val half was significantly less abundant in colorectal cancer EVs compared to the tRNA Gly half, and it promoted the production of inflammatory cytokines by macrophages in a TLR8‐dependent manner (Pawar et al. 2020). It is intriguing that the tRNA halves encapsulated in EVs activate different TLRs as their targets. However, the mechanism of their preferential loading into EVs remains unknown. Our small RNA‐seq analysis clearly showed that 5'‐half and 5'‐tRF make up a large portion of the population within the EVs. If tRNA fragmentation were occurring within EVs, we would expect other tRNA fragments to appear in similar proportions to the 5'‐half and 5'‐tRF. Therefore, there may be a mechanism that selectively sorts tRNA fragments into EVs.

Experiments with TLR3 inhibitor CU‐CPT‐4a and TLR3 siRNA suggested a negligible contribution of TLR3. However, it is possible that TLR3 is also involved in the inflammatory cytokine production‐promoting mechanism induced by colorectal cancer EVs. Toll‐like receptor 3 (TLR3) is a member of the TLR family, which is responsible for recognising viral double‐stranded RNA (dsRNA). Since 5′‐half‐GlyGCC have been reported to form dimers (Tosar et al. 2018), it is possible that some of the tRNA halves within the EVs may form dimers, thereby interacting with TLR3.

## Author Contributions

**Yuya Monoe**: data curation (equal), formal analysis (equal), funding acquisition (equal), investigation (equal), methodology (equal), validation (equal), visualization (equal), writing – original draft (equal), writing – review and editing (equal). **Kentaro Jingushi**: conceptualization (equal), data curation (equal), formal analysis (equal), funding acquisition (equal), investigation (equal), methodology (equal), project administration (equal), validation (equal), visualization (equal), writing – original draft (equal), writing – review and editing (equal). **Kohei Taniguchi**: data curation (equal), investigation (equal), methodology (equal), writing – review and editing (equal). **Kensuke Hirosuna**: data curation (equal), formal analysis (equal), investigation (equal), methodology (equal). **Jun Arima**: investigation (equal), methodology (equal). **Yosuke Inomata**: investigation (equal), methodology (equal). **Yoshiaki Takano**: investigation (equal), methodology (equal). **Hiroki Hamamoto**: investigation (equal), methodology (equal). **Kazumasa Komura**: data curation (equal), investigation (equal), methodology (equal). **Tomohito Tanaka**: data curation (equal), investigation (equal), methodology (equal). **Hiroaki Hase**: investigation (equal), methodology (equal). **Sang‐Woong Lee**: funding acquisition (equal), investigation (equal), supervision (equal), writing – original draft (equal), writing – review and editing (equal). **Kazutake Tsujikawa**: funding acquisition (equal), investigation (equal), supervision (equal), writing – original draft (equal), writing – review and editing (equal).

## Conflicts of Interest

The authors declare no conflicts of interest.

## Supporting information



Supporting Information

Supporting Information

Supporting Information

Supporting Information

Supporting Information

Supporting Information

Supporting Information
