# Supplementary material for: Cancer‐Specific RNA Modifications in Tumour‐Derived Extracellular Vesicles Promote Tumour Growth
Source: J Extracell Vesicles. 2025 May 6;14(5):e70083. doi: 10.1002/jev2.70083 (PMC12053886; doi:10.1002/jev2.70083)

Supplementary Materials for

**Decreased N6-methyladenosine levels on 5′-half-GlyGCC in tumor-derived extracellular vesicles promote tumor growth via TLR8 in macrophages**

Yuya Monoe *et al.*

*Corresponding author. Email: jingushi-kk@phs.osaka-u.ac.jp

**This file includes:**

Figs. S1 to S12

Relevant certificates

**
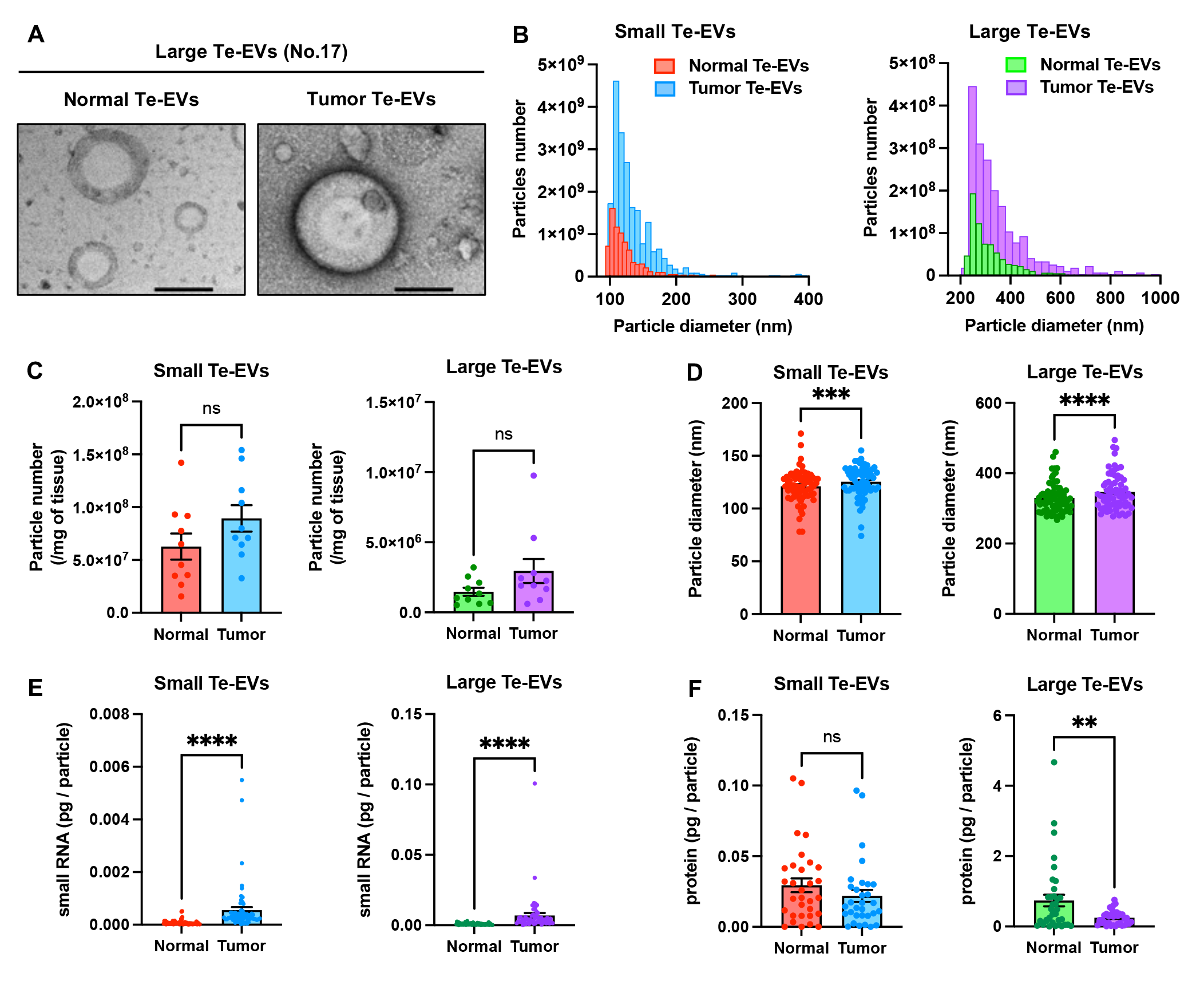
**

Fig. S1. Characterization of normal and tumor Te-EVs, related to Figure 1.

**(A)** Representative image of non-cancerous colon large Te-EVs (Normal Te-EVs) and colorectal cancer large Te-EVs (Tumor Te-EVs). Black bars indicate 200 nm. (**B)** Particle distributions of small and large Te-EVs. (**C)** Number of small and large Te-EVs per unit tissue weight (n = 10). Values are presented as the mean ± SEM for each group. Wilcoxon signed-rank test; ns: not significant. (**D)** Particle diameters of small (n = 66) and large (n = 72) Te-EVs. Values are presented as the mean ± SEM for each group. Wilcoxon signed rank test; ***P < 0.001, ****P < 0.0001. (**E)** Amounts of small RNA in small (n = 31) and large (n = 27) Te-EVs. Values are presented as the mean ± SEM for each group. Wilcoxon signed-rank test; ****P < 0.0001. (**F)** Protein levels in small (n = 31) and large (n = 36) Te-EVs. Values are presented as the mean ± SEM for each group. Wilcoxon signed-rank test; **P < 0.01, ns: not significant.


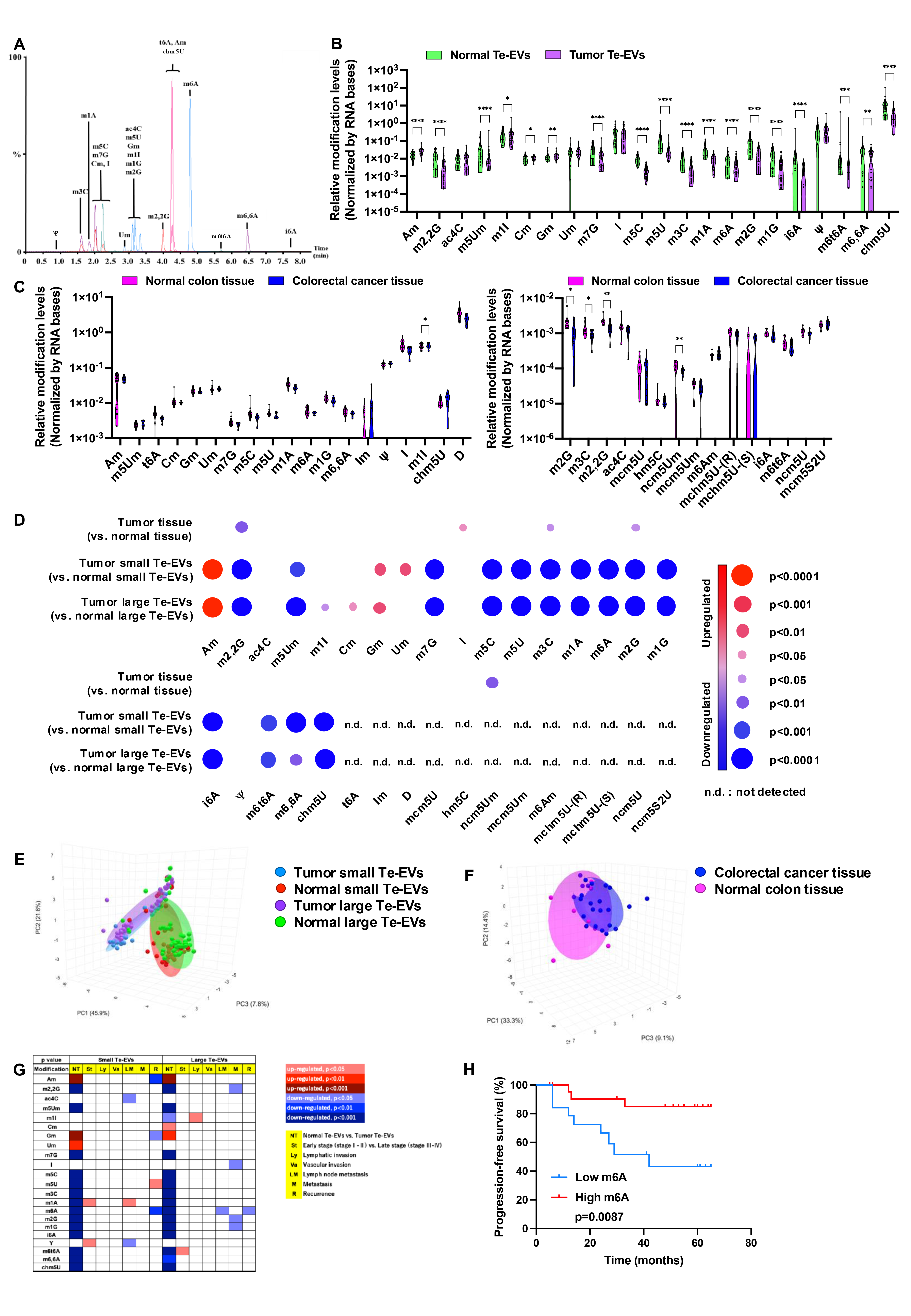


Fig. S2. Characterization of RNA modification profiles in Te-EVs and corresponding tissues, related to Figure 1.

**(A)** Peak images of each RNA modification successfully detected using EV-RNA. (**B)** UHPLC-MS/MS results for normal large Te-EVs (n = 48) and large tumor Te-EVs (n = 48). Wilcoxon signed rank test; *P < 0.05, **P < 0.01, ***P < 0.001, ****P < 0.0001. (**C)** UHPLC-MS/MS results for normal (n =8) and tumor tissues (n =24). Mann–Whitney test; *P < 0.05, **P < 0.01. (**D)** Schematic comparison of various RNA modifications in tumor tissue versus normal tissue, tumor small Te-EVs versus normal small Te-EVs, and tumor large Te-EVs versus normal large Te-EVs. Red circles indicate increased RNA modification levels and blue circles indicate decreased RNA modification levels. The size of the circle indicates the degree of the P-value (Mann–Whitney U test). ns: not significant. Principal component analysis plot of RNA modification data in Te-EVs (**E**) and corresponding tissues (**F**). (**G)** Comparison of RNA modifications detected in EVs in various clinical backgrounds. (**H**) Progression-free survival of colorectal cancer patients who had low (n=19) or high (n=23) m6A levels in tumor-derived small Te-EVs. Log-rank test.


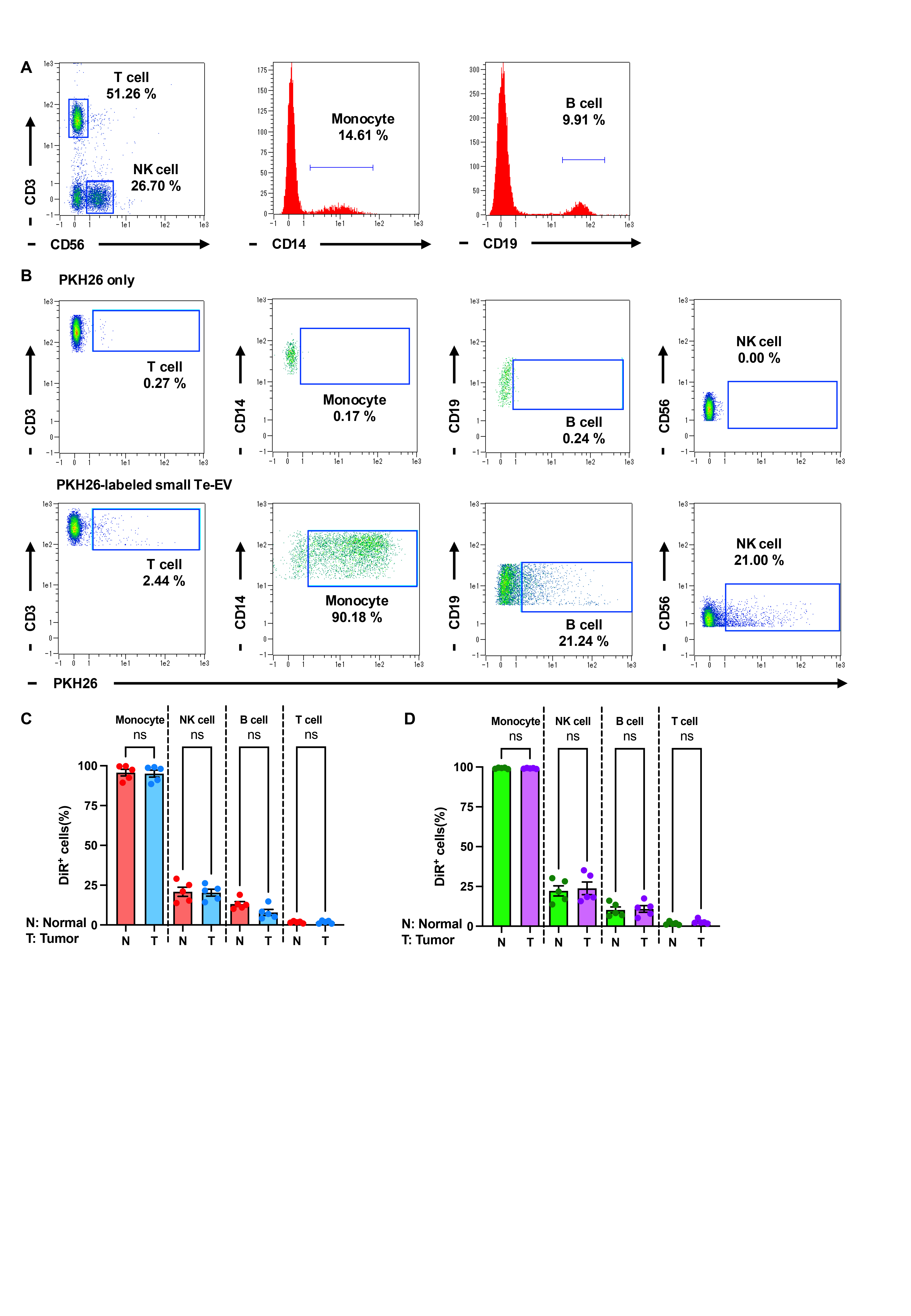


**Fig. S3. Monocytes exhibited the highest uptake of small and large Te-EVs among PBMCs, related to Figure 2.**

PBMCs were pretreated with PKH26-labeled Te-EVs. (A) The percentage of each cell type and (B) PKH26-positive EVs in PBMCs were analyzed via flow cytometry. A representative image is shown. (C) Normal colon small EVs (n=5) and colon cancer tissue small EVs (n=5) were labeled with DiR and added to PBMCs. The percentage of DiR-positive EVs in PBMCs was determined via flow cytometry. Values are presented as the mean ± SEM for each group. Wilcoxon signed-rank test; ns: not significant. (D) Normal colon large EVs (n=5) and colon cancer tissue large EVs (n=5) were labeled with DiR and added to PBMCs. The percentage of DiR-positive EVs among each PBMC type was measured via flow cytometry. Values are presented as the mean ± SEM for each group. Wilcoxon signed-rank test; ns: not significant.


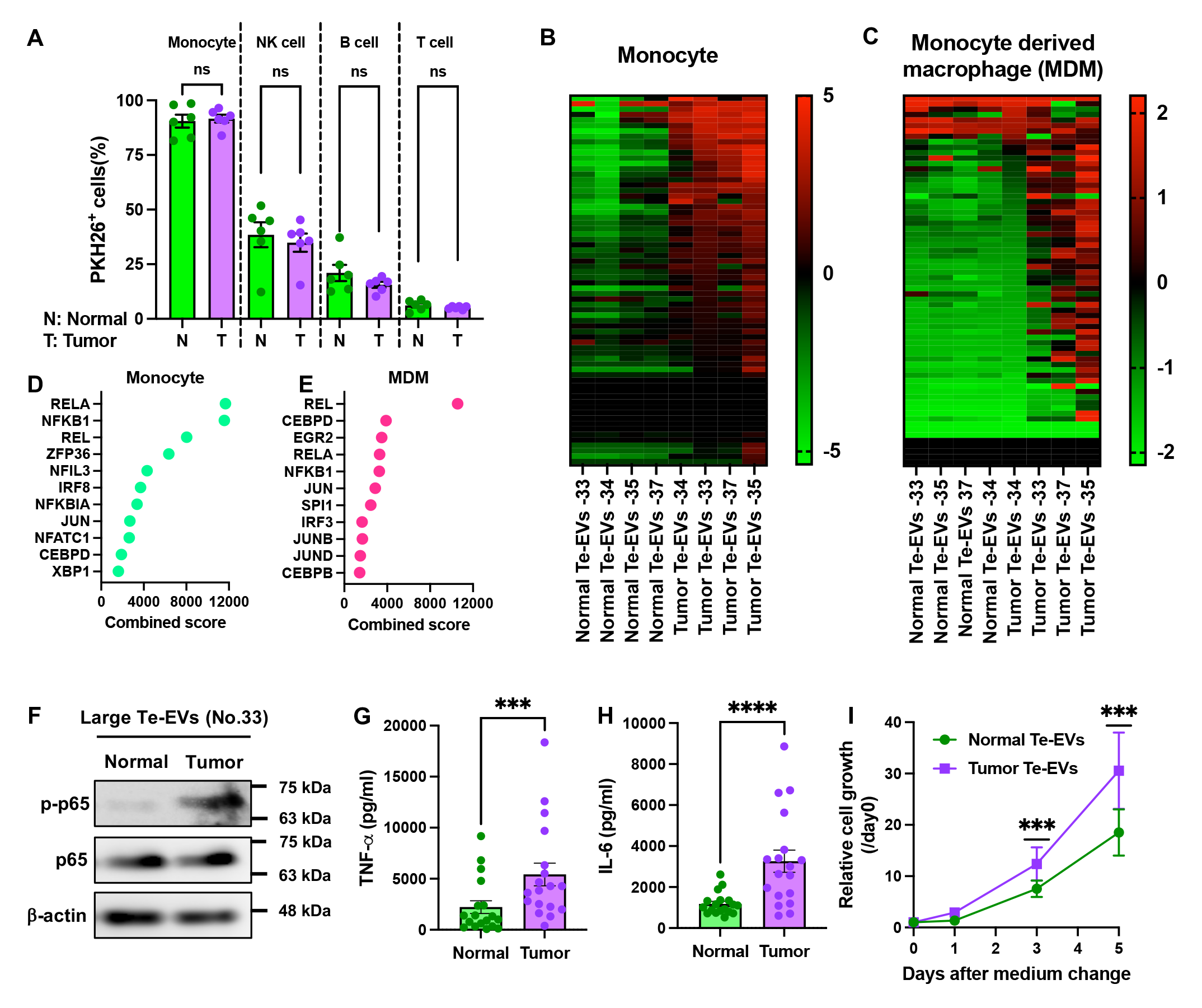


Fig. S4. Colorectal cancer large Te-EVs promote inflammation in macrophages and cancer cell proliferation related to Figure 2.

**(A)** Normal colon large EVs (n=6) and colon cancer tissue large EVs ( n=6) were labeled with PKH26 and added to PBMCs. The percentage of PKH26-positive EVs in each PBMC was measured via flow cytometry. Values are presented as the mean ± SEM for each group. Wilcoxon signed-rank test; ns: not significant. B, Heatmap of the cytokine array using conditioned medium from monocytes (**B**) or monocyte-derived macrophages (MDM, **C**) treated with normal (n = 4) or tumor (n = 4) large Te-EVs. Enrichment analysis of the cytokine array using conditioned medium from monocytes (**D**) or MDMs (**E**) treated with normal (n = 4) or tumor (n = 4) large Te-EVs. (**F)** Whole-cell lysates obtained from normal or tumor large Te-EV-treated MDM were analyzed by western blotting using anti-phospho p65, anti-p65, and anti-β-actin antibodies. A representative image of three independent experiments is shown. ELISA was conducted using conditioned media from MDMs treated with normal (n = 18) or tumor (n = 18) large Te-EVs. TNF-α (**G**) and IL-6 (**H**) levels. Values are presented as the mean ± SEM for each group. Wilcoxon signed-rank test; ***P < 0.001, ****P < 0.0001. (**I)** HT29 cells were cultured in conditioned medium from MDMs treated with normal (n = 18) or tumor (n = 18) large Te-EVs. Values are presented as the mean ± SEM for each group. Mann–Whitney U test; ***P < 0.001.

**
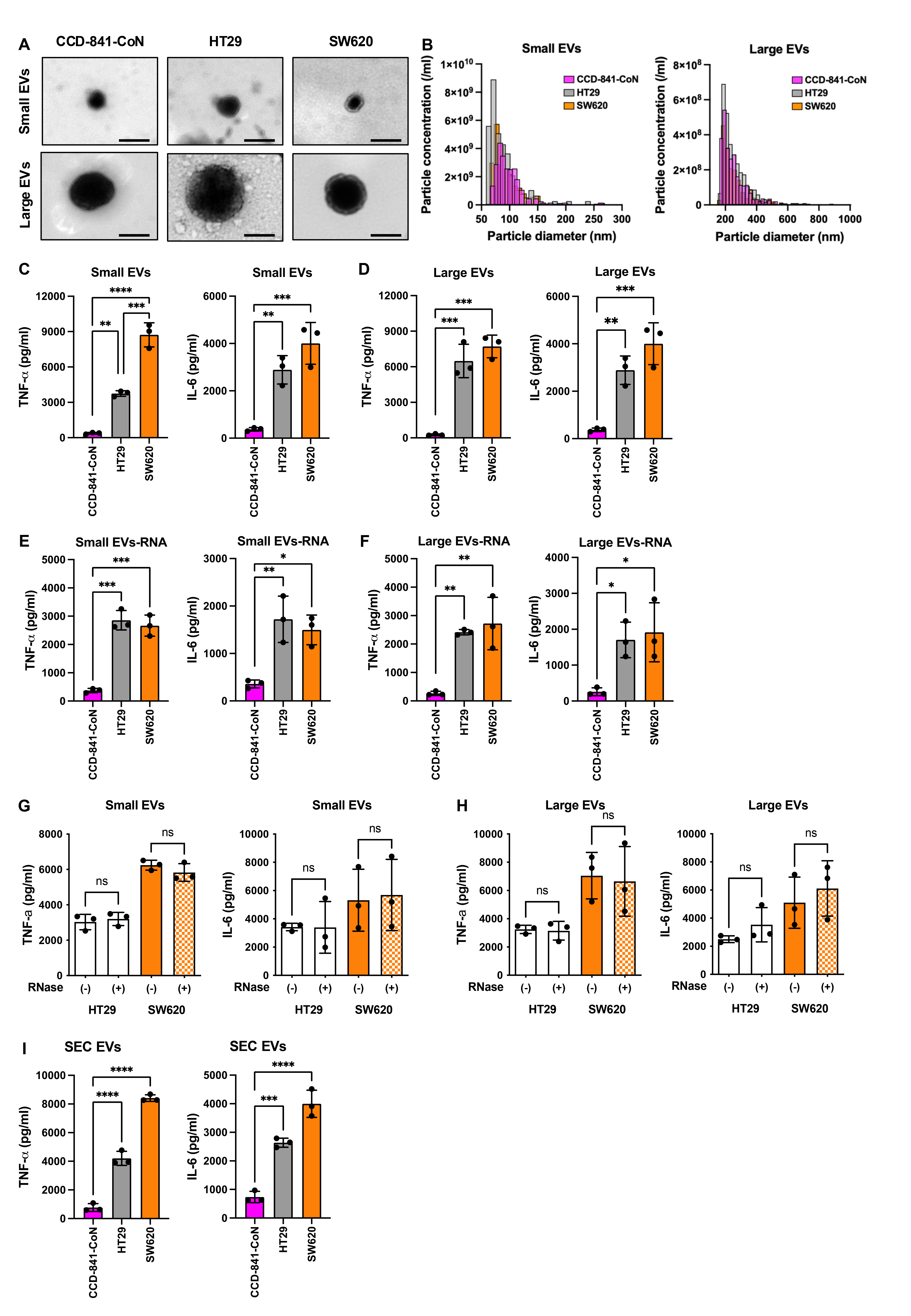
**

**Fig. S5. Colorectal cancer cell line-derived EVs promote TNF-α and IL-6 secretion by macrophages, related to Figure 2.**

**(A)** Representative images of CCD-841-CoN, HT29, and SW620 EVs. Black bars indicate 200 nm. **(B)** Particle distribution of small and large cell line-derived EVs. ELISA was conducted using conditioned medium from differentiated THP-1 cells (dTHP-1) treated with small cell line-derived EVs. TNF-α **(C, left)** and IL-6 **(C, right)** concentration. Values are presented as the mean ± SEM for each group. One-way ANOVA post hoc Tukey’s test; **P < 0.01, ***P < 0.001, ****P < 0.0001. ELISA was conducted using conditioned medium from dTHP-1 cells treated with cell line-derived large EVs. TNF-α **(D, left)** and IL-6 **(D, right)** concentration. Values are presented as the mean ± SEM for each group. One-way ANOVA post hoc Tukey’s test; **P < 0.01, ***P < 0.001. ELISA was conducted using conditioned medium from dTHP-1 cells transfected with cell line-derived small EVs-RNA. TNF-α **(E, left)** and IL-6 **(E, right)** concentration. Values are presented as the mean ± SEM for each group. One-way ANOVA post hoc Tukey’s test; *P < 0.05, **P < 0.01, ***P < 0.001. ELISA was conducted using conditioned medium from dTHP-1 cells transfected with cell line-derived large EVs-RNA. TNF-α **(F, left)** and IL-6 **(F, right)** concentration. Values are presented as the mean ± SEM for each group. One-way ANOVA post hoc Tukey’s test; *P < 0.05, **P < 0.01. Cell line-derived EVs were pretreated with or without RNase. ELISA was conducted using conditioned medium from dTHP-1 cells treated with small cell line-derived EVs. TNF-α **(G, left)** and IL-6 **(G, right)** concentration. Values are presented as the mean ± SEM for each group. Unpaired t-test; ns: not significant. Cell line-derived EVs were pretreated with or without RNase. ELISA was conducted using conditioned medium from dTHP-1 cells treated with cell line-derived large EVs. TNF-α **(H, left)** and IL-6 **(H, right)** concentration. Values are presented as the mean ± SEM for each group. Unpaired t-test; ns: not significant. Cell line-derived EVs were purified via size exclusion chromatography. ELISA was conducted using conditioned medium from dTHP-1 cells treated with purified small cell line-derived EVs. TNF-α **(I, left)** and IL-6 **(I, right)**

**
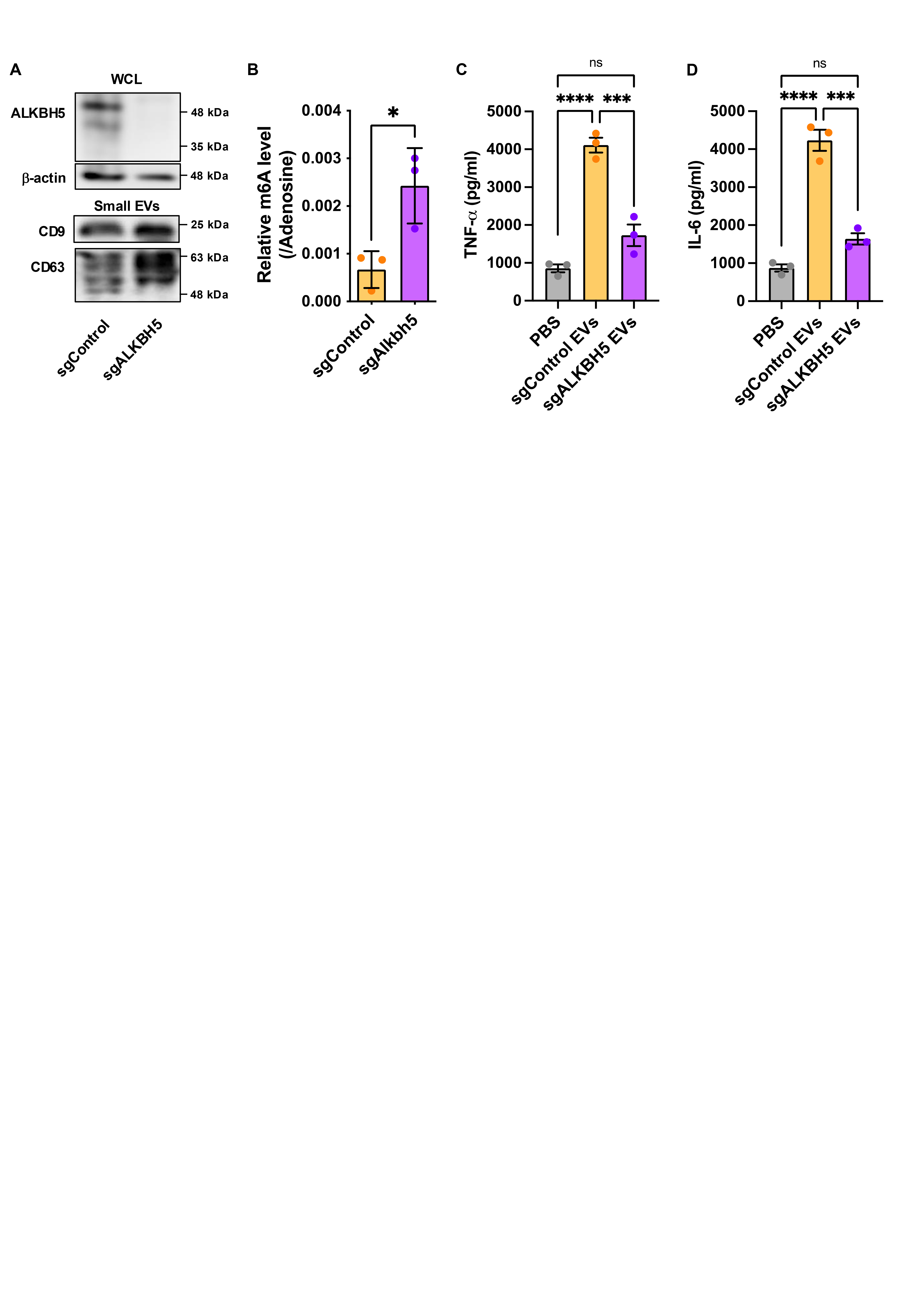
**

**Fig. S6. Upregulation of m6A levels in colorectal cancer cell line-derived EVs impeded the inflammatory response in macrophages, related to Figure 2.**

(A) Whole-cell lysates and small EVs obtained from control HT29 cells (sgControl) or ALKBH5 knockout HT29 cells (sgALKBH5) were subjected to western blot analysis using anti-ALKBH5, anti-β-actin, anti-CD9, and anti-CD63 antibodies. Representative images from three independent experiments are shown. (B) UHPLC-MS/MS results for sgControl and sgALKBH5 EVs. Values are presented as the mean ± SEM for each group. Unpaired t-test; *P < 0.05. ELISA was conducted using conditioned medium from dTHP-1 cells treated wth sgControl or sgALKBH5 HT29 small EVs. TNF-α **(C)** and IL-6 **(D)** concentration. Values are presented as the mean ± SEM for each group. One-way ANOVA with Tukey’s post hoc test; ***P < 0.001, ****P < 0.0001, ns: not significant.


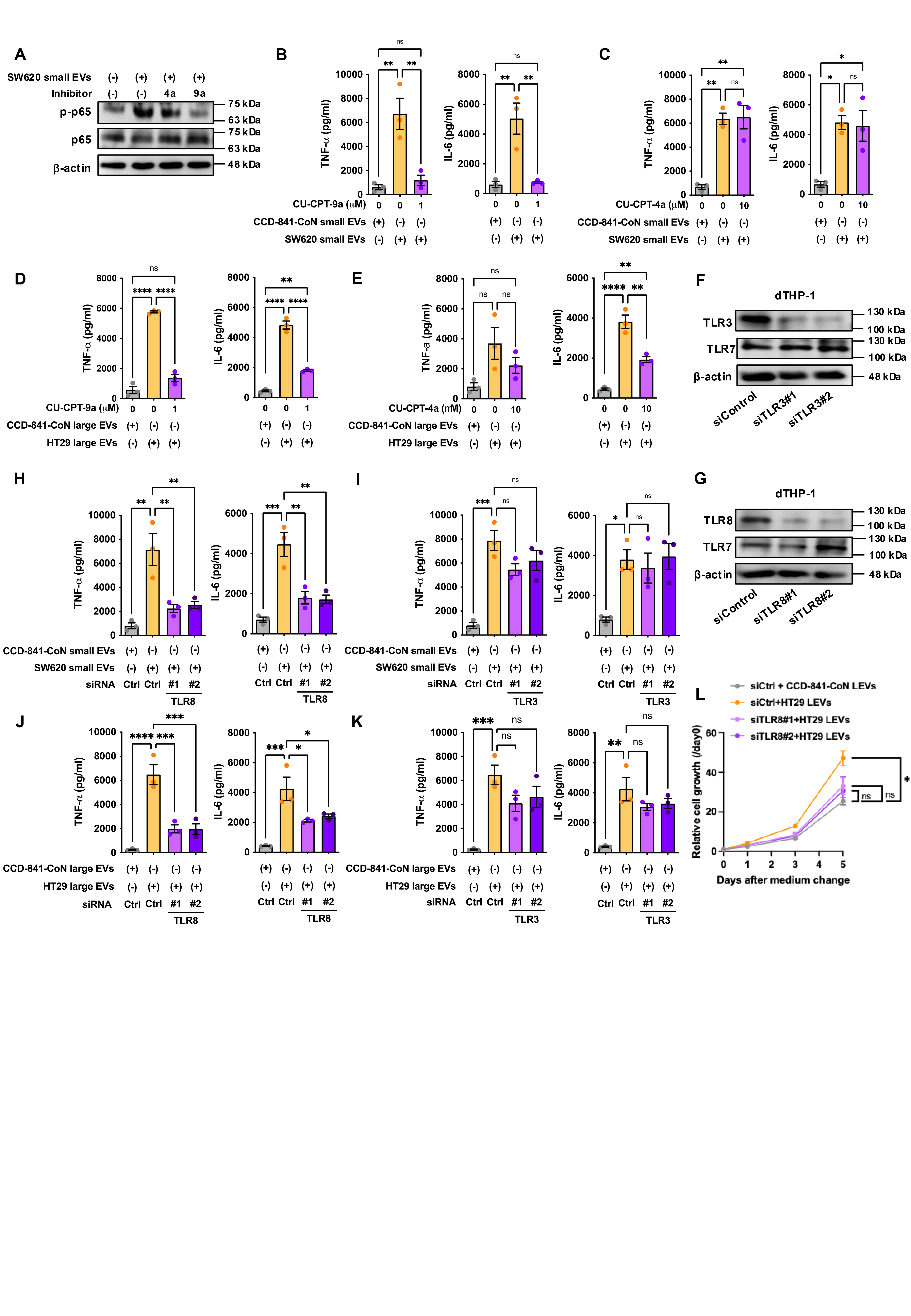


Fig. S7. Tumor-derived large EVs regulate inflammatory responses in macrophages via TLR8, related to Figure 4.

Differentiated THP-1 cells (dTHP-1) were pretreated with or without CU-CPT-9a and CU-CPT-4a. (A) Whole-cell lysates obtained from dTHP-1 cells treated with SW620 small EVs were subjected to western blot analysis using an anti-phospho p65 antibody, anti-p65 antibody, and anti-β-actin antibody. Representative images from three independent experiments are shown. Conditioned medium from dTHP-1 cells treated with SW620 small EVs was used for ELISA. TNF-α (**B, left and C, left**) and IL-6 (B, right and **C, right**). Values are presented as the mean ± SEM for each group. Wilcoxon signed-rank test; *P < 0.05, **P < 0.01, ns: not significant. Conditioned medium from dTHP-1 cells treated with CCD-841-CoN or HT29 large EVs was used for ELISA. TNF-α (**D, left and E, left**) and IL-6 (D, right and **E, right**). Values are presented as the mean ± SEM for each group. One-way ANOVA with post hoc Tukey’s test; **P < 0.01, ****P < 0.001, ns: not significant. Whole-cell lysates obtained from dTHP-1 cells transfected with TLR3 (**F**) or TLR8 (**G**) siRNAs were analyzed via western blotting using anti-TLR3, anti-TLR8, anti-TLR7, and anti-β-actin antibodies. Representative images from three independent experiments are shown. dTHP-1 cells were pre-transfected with or without TLR8 siRNA. Conditioned medium containing dTHP-1 cells treated with SW620 small EVs was used for ELISA. TNF-α (**H, left**), and IL-6 (**H, right**). Values are presented as the mean ± SEM for each group. One-way ANOVA post hoc Tukey’s test; **P < 0.01, ***P < 0.001. dTHP-1 cells were pre-transfected with or without TLR3 siRNA. Conditioned medium containing dTHP-1 cells treated with SW620 small EVs was used for ELISA. TNF-α (**I, left**), and IL-6 (**I, right**). Values are presented as the mean ± SEM for each group. One-way ANOVA post hoc Tukey’s test; *P < 0.05, ***P < 0.001, ns: not significant. dTHP-1 cells were pre-transfected with or without TLR8 siRNA. Conditioned medium containing dTHP-1 cells treated with CCD-841-CoN or HT29 large EVs was used for ELISA. TNF-α (**J, left**), and IL-6 (**J, right**). Values are presented as the mean ± SEM for each group. One-way ANOVA post hoc Tukey’s test; *P < 0.05, ***P < 0.001, ****P < 0.0001. dTHP-1 cells were pre-transfected with or without TLR3 siRNA. Conditioned medium containing dTHP-1 cells treated with CCD-841-CoN large EVs or HT29 large EVs was used for ELISA. TNF-α (**K, left**), and IL-6 (**K, right**). Values are presented as the mean ± SEM for each group. One-way ANOVA post hoc Tukey’s test; **P < 0.01, ***P < 0.001, ns: not significant. (**L)** HT29 cells were cultured in conditioned medium containing dTHP-1 cells transfected with or without TLR8 siRNA and CCD-841-CoN or HT29 large EVs. Values are presented as the mean ± SEM for each group. One-way ANOVA post hoc Tukey’s test; *P < 0.05, ns: not significant.


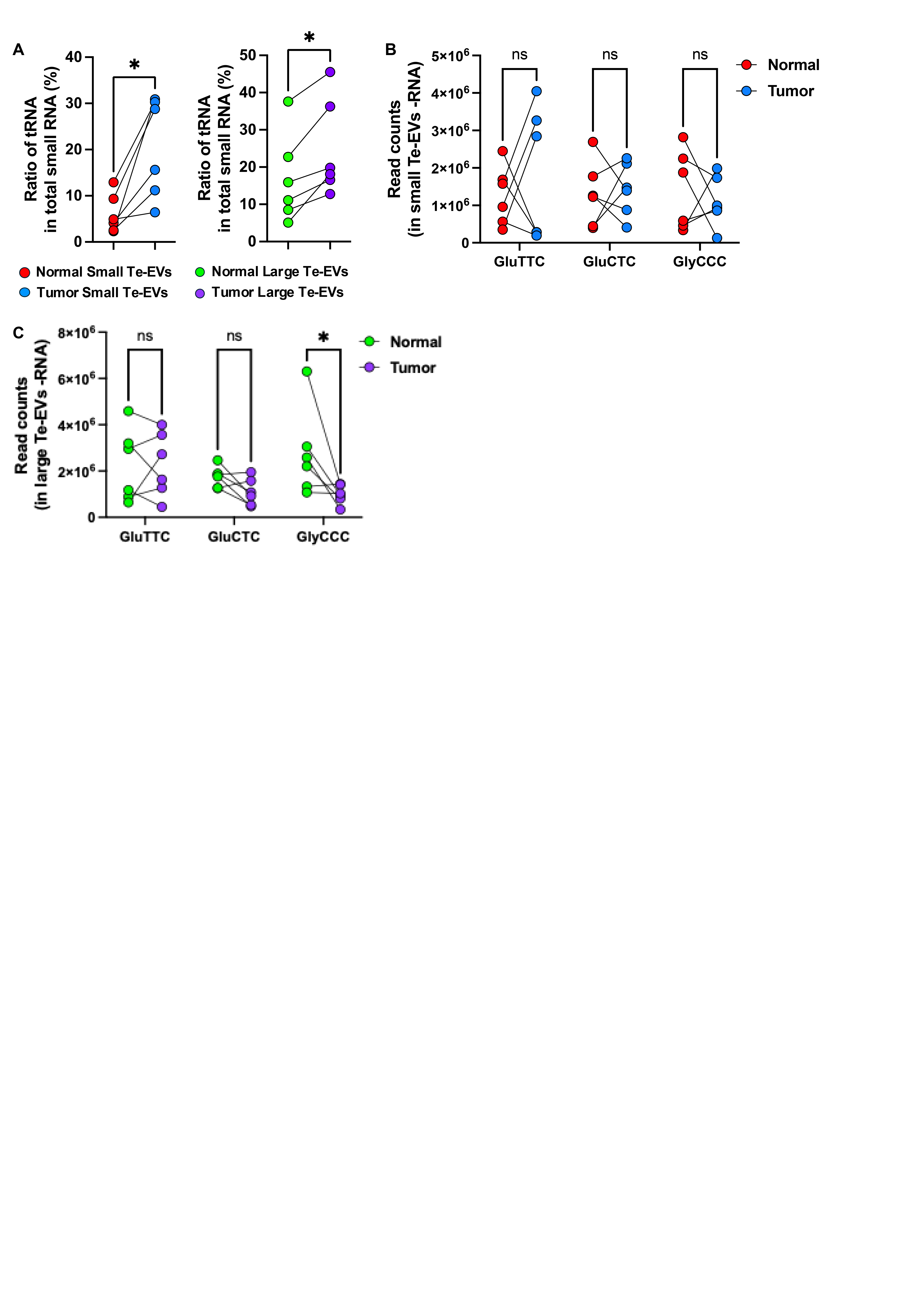


Fig. S8. Transfer RNA-derived fragment analysis of small RNA-seq data in Te-EVs, related to Figure 5.

**(A)** Ratio of tRNA in normal (n = 6) and tumor (n = 6) small and large Te-EVs from small RNA-seq data. (**B)**Read counts of 5′-half-Glu-TTC, 5′-half-Glu-CTC, and 5′-half-GlyCCC in normal (n = 6) and tumor (n = 6) small Te-EVs. (**C)** Read counts of 5′-half-Glu-TTC, 5′-half-Glu-CTC, and 5′-half-GlyCCC in normal (n = 6) and tumor (n = 6) large Te-EVs.


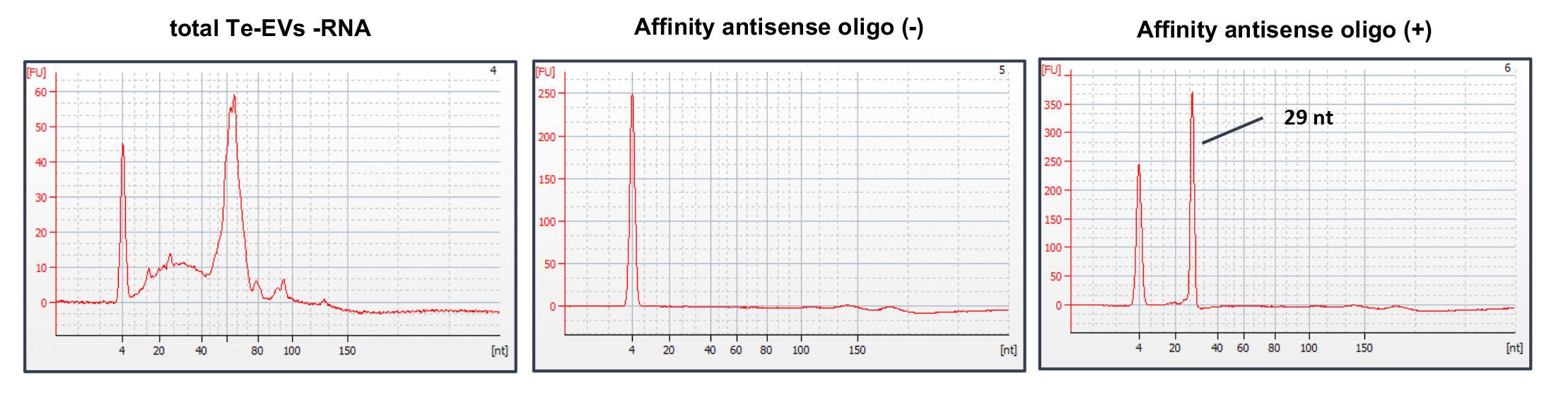


Fig. S9. Electrophoresis image of 5′-half-GlyGCC pull-down, related to Figure 5.

**(A)** Electrophoresis image of Te-EV-RNA and pulled-down 5′-half-GlyGCC Te-EV -RNA obtained using a Bioanalyzer. Representative images of seven independent experiments are shown.

**
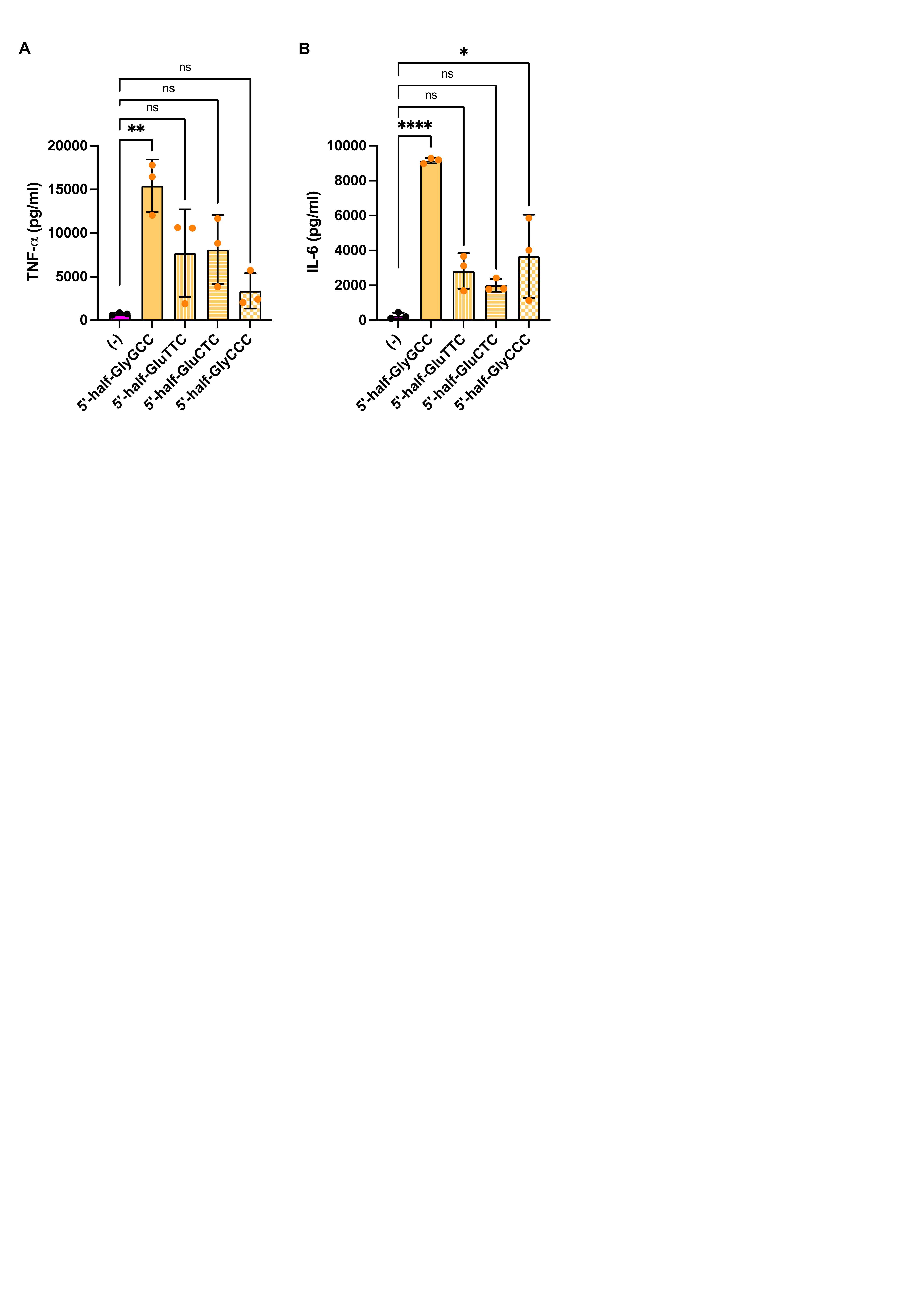
**

Fig. S10. Effect of 5’-halves on inflammatory cytokine secretion in dTHP-1 cells, related to Figure 6.

Differentiated THP-1 cells (dTHP-1) were transfected with 5′-half-GlyGCC, 5′-half-GluTTC, 5′-half-GluCTC, or 5′-half-GlyCCC. Conditioned medium from dTHP-1 cells was used for ELISA. TNF-α (**A**) and IL-6 (**B**). Values are presented as the mean ± SEM for each group. One-way ANOVA post hoc Tukey’s test; *P<0.05, **P < 0.01, ****P < 0.0001, ns: not significant.


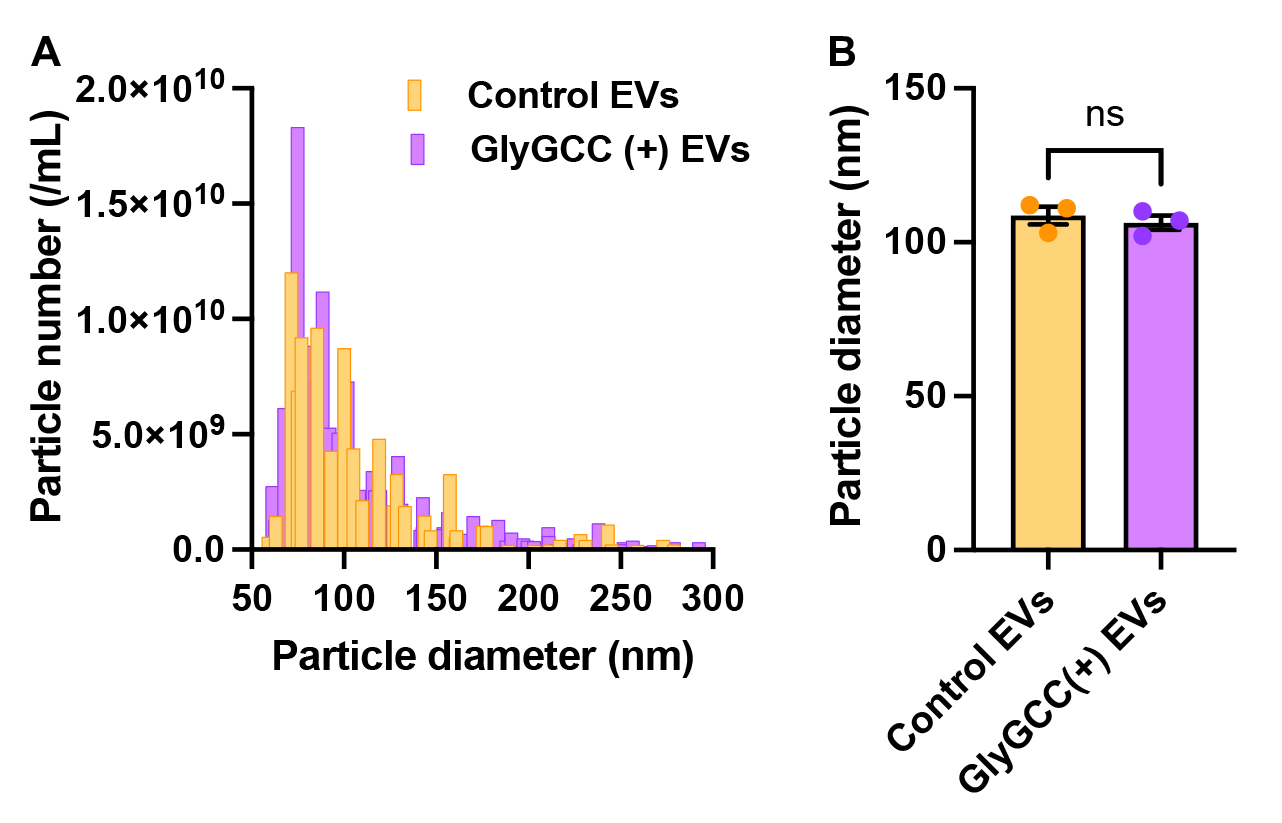


Fig. S11. Characterization of 5′-half-GlyGCC-loaded colon 26 small EVs, related to Figure 6.

**(A)** Particle distribution of 5′-half-GlyGCC-loaded colon26 small EVs (GlyGCC(+) EVs) and control colon26 EVs. **(B)** Particle diameters of GlyGCC(+) and Control EVs. Values are presented as the mean ± SEM for each group. Unpaired t-test; ns: not significant.

**
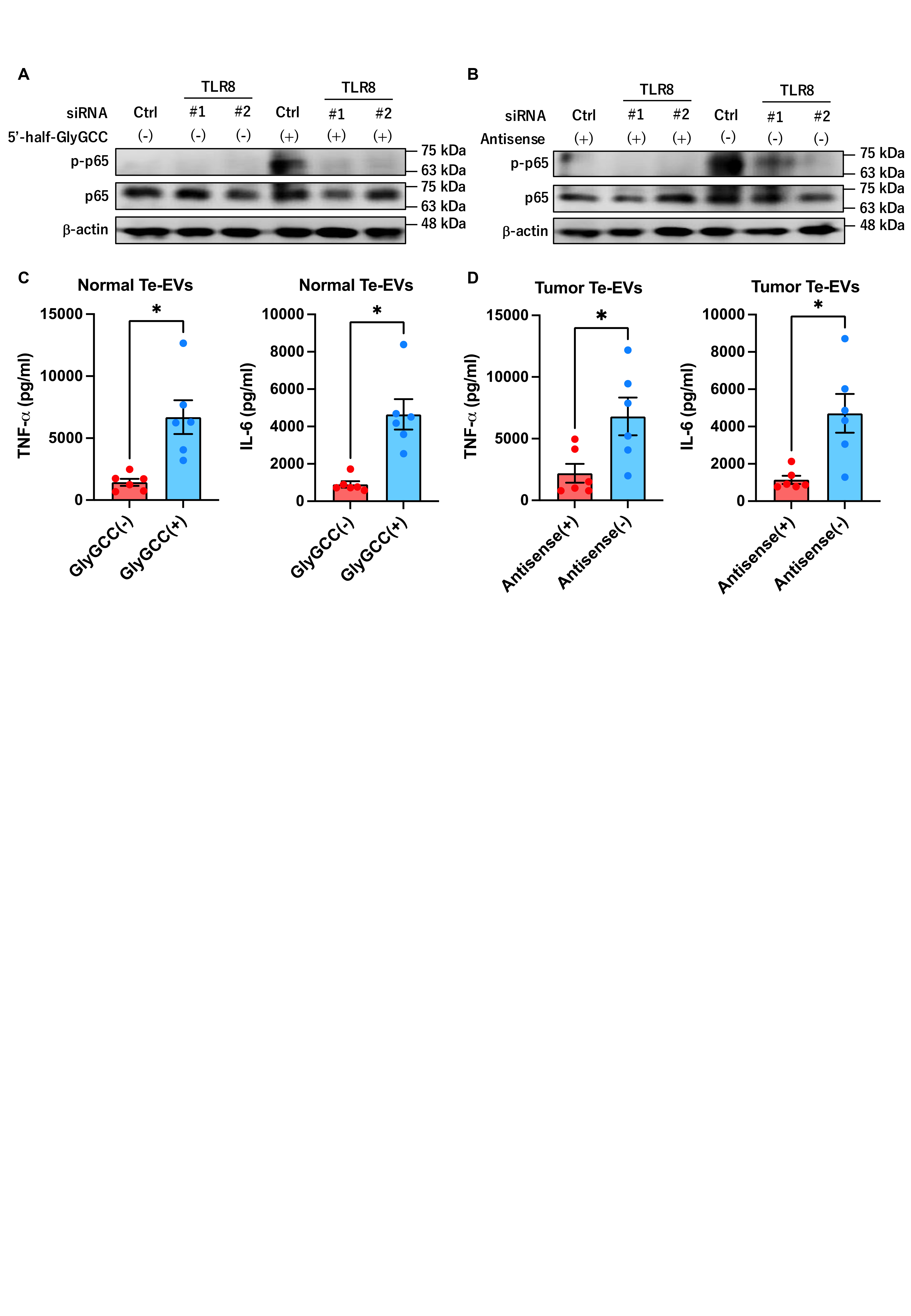
**

**Fig. S12. A 5’-half-GlyGCC antisense oligo abrogated the TLR8-mediated inflammatory response induced by tumor-derived Te-EVs, related to Figure 6.**

dTHP-1 cells were pre-transfected with or without TLR8 siRNA. (A) Whole-cell lysates obtained from dTHP-1 cells treated with 5’-half-GlyGCC-loaded normal Te-EVs were subjected to western blot analysis using an anti-phospho p65 antibody, anti-p65 antibody, and anti-β-actin antibody. Representative images from three independent experiments are shown. (B) Whole-cell lysates obtained from dTHP-1 cells treated with 5’-half-GlyGCC antisense-loaded tumor Te-EVs were subjected to western blot analysis using an anti-phospho p65 antibody, anti-p65 antibody, and anti-β-actin antibody. Representative images from three independent experiments are shown. ELISA was conducted using conditioned medium from dTHP-1 cells treated with 5’-half-GlyGCC-loaded normal Te-EVs. TNF-α (C, left) and IL-6 (C, right) concentration. Values are presented as the mean ± SEM for each group. Wilcoxon signed-rank test; *P < 0.05. ELISA was conducted using conditioned medium from dTHP-1 treated with 5’-half-GlyGCC antisense-loaded tumor Te-EVs. TNF-α (D, left) and IL-6 (D, right) concentration. Values are presented as the mean ± SEM for each group. Wilcoxon signed-rank test; *P < 0.05.

**Cell lines authentication**

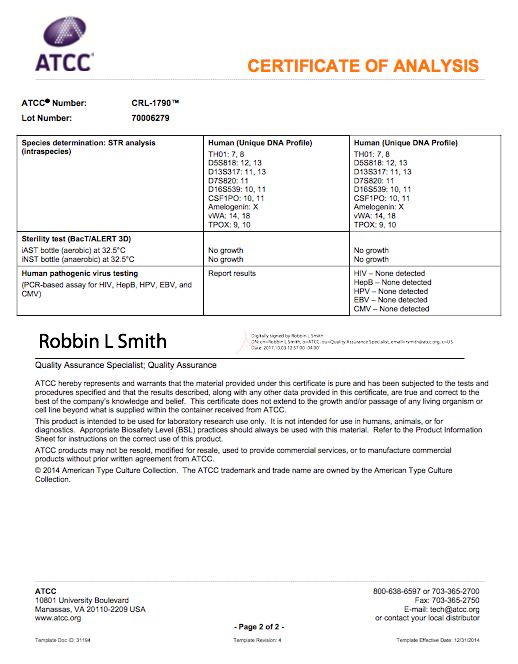


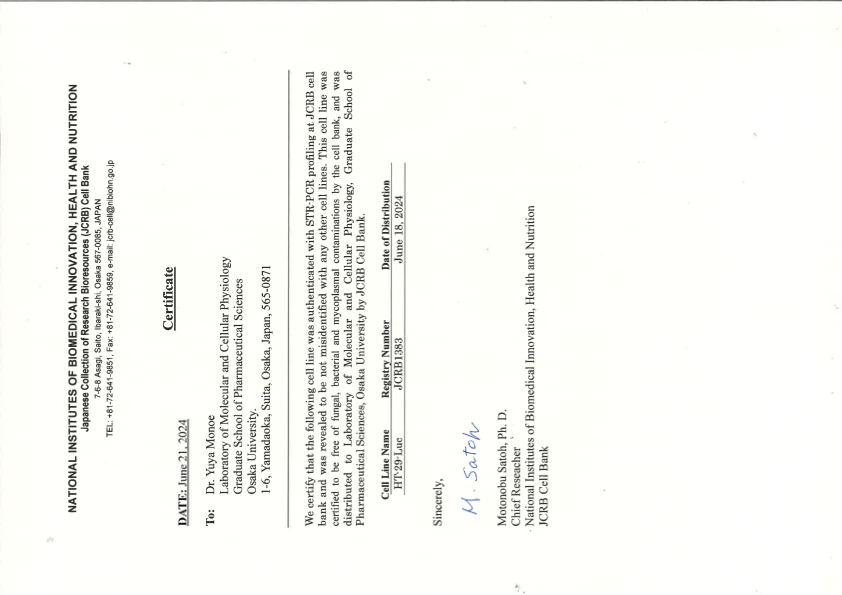

Supplement: Supplementary file 1 — Supporting Information [file JEV2-14-e70083-s001.docx]
